# Study protocol: the OxWell school survey investigating social, emotional and behavioural factors associated with mental health and well-being

**DOI:** 10.1136/bmjopen-2021-052717

**Published:** 2021-11-30

**Authors:** Karen Laura Mansfield, Stephen Puntis, Emma Soneson, Andrea Cipriani, Galit Geulayov, Mina Fazel

**Affiliations:** 1 Department of Psychiatry, University of Oxford, Oxford, UK; 2 Department of Psychiatry, University of Cambridge, Cambridge, UK; 3 Oxford Health NHS Foundation Trust, Warneford Hospital, Oxford, UK

**Keywords:** mental health, child & adolescent psychiatry, epidemiology

## Abstract

**Introduction:**

Improving our understanding of the broad range of social, emotional and behavioural factors that contribute to mental health outcomes in adolescents will be greatly enhanced with diverse, representative population samples. We present a protocol for a repeated self-report survey assessing risk and protective factors for mental health and well-being in school pupils aged 8–18 years with different socioeconomic backgrounds in England. The survey will provide a comprehensive picture of mental health and associated risks at the community level to inform the development of primary and secondary prevention and treatment strategies in schools.

**Methods and analysis:**

This protocol is for a large-scale online repeated self-report survey, representative of children and adolescents aged 8–18 years attending schools or further education colleges in participating counties in England. The survey consists of around 300 questions, including validated measures of mental health and well-being, risk and protective factors, and care-seeking behaviour and preferences. Additional questions each year vary to address current events and novel hypotheses, developed by the research team, collaborators and stakeholders. Primary analyses will investigate current and changing risk and protective factors, care-seeking behaviour and attitudes to allowing linkage of their sensitive data to other databases for research, and will compare measures of mental health to measures of well-being.

**Ethics and dissemination:**

The study was approved by the University of Oxford Research Ethics Committee (Reference: R62366). Tailored data summaries will be provided to participating schools and stakeholders within 3 months of data collection. The main findings will be presented at scientific meetings, published in peer-reviewed journals and shared via digital and social media channels. At the end of the study, other researchers will be able to apply for access to anonymous data extracts.

**Strengths and limitations of this study**

- This survey measures current and sensitive risk factors, and attitudes to care seeking and data sharing, in a large and diverse sample of school pupils in England.
- The work involves considerable stakeholder involvement including local authorities, schools and young people.
- The survey is repeated cross-sectional in design and analyses can therefore provide only limited insight into causality.
- Differences in data collection methods between schools and survey years may influence representativeness.
- The most sensitive questions around mental health and risk factors cannot be asked to younger pupils and alternative measures are not always comparable.

## Introduction

The importance of understanding the range of social, emotional and behavioural factors that contribute to the mental health of children and adolescents is becoming increasingly evident, and such insight at the community and population level can best inform promotion, prevention and intervention strategies.^1^ There is evidence of increasing rates of emotional disorders in children and adolescents, with the poorest mental health among adolescents of secondary school age.^2–4^ This has been exacerbated by the additional pressures the COVID-19 pandemic has placed on this population.^5^ A comprehensive method is needed to examine the many interconnected factors that might be influencing adolescent mental health and well-being. For example, adolescents were already spending more time using digital media and participating less in physical activity,^6 7^ which is associated with poorer mental well-being in this group.^8^ Not enough is known about how these and other changing lifestyle factors relate to the mental health of children and adolescents, for example, with contrasting findings in relation to the role of social media.^9^ School and population based surveys are increasingly being used to build an evidence base to understand school pupils’ well-being, which could learn from initiatives to identify appropriate indicators of mental health difficulties at the population level.^10–15^ However, more evidence is needed to accurately quantify rapidly changing risk factors, and the protective factors that might mitigate them, to inform school-based interventions.

This paper presents the OxWell Student Survey, a study and analysis protocol for an online repeated cross-sectional survey. OxWell is an adaptation of an existing survey first conducted biannually for over a decade in schools in one local authority with more recent adoption by neighbouring local authorities in England.^16 17^ The original survey was conceived by public health practitioners seeking better insight into the needs and behaviours of school pupils in their area (eg, in relation to self-harm).^18^ The OxWell study team began collaborating with the survey provider, schools, and local authorities in 2019 and collected baseline data on mental well-being in 2019 and 2020. This protocol describes adaption of the survey in 2021 with the aim to attain a comprehensive picture of child and adolescent mental health, current risk factors and opinions regarding mental health support in a school-aged (8–18 years) population sample.

### Objectives

The goals for the survey are:

- To investigate prevalence and variability in pupil-reported mental health and well-being.
- To measure population-level changes in school pupils’ mental health, social vulnerability and risk-taking behaviours across survey years.
- To investigate pupil’s attitudes towards and experience of seeking mental health support.
- To identify opportunities for developing and evaluating school-based mental health promotion, prevention and treatment interventions.
- To understand young people’s views on allowing linkage of their survey data to other databases for research purposes.

## Methods and analysis

### Study design

The OxWell Student Survey is a repeated cross-sectional survey targeted at school-aged children and adolescents (8–18 years) in England. The data are collected during school-time, without collecting names, addresses or date of birth of pupils, or using unique logins. This is intended to encourage open and accurate responses, including some sensitive questions on vulnerability, risk-taking behaviour and social risk factors (eg, domestic abuse), while protecting participants’ privacy. Due to data protection and ethical requirements, exact measures on ethnicity, gender identity and disability cannot be asked, but some demographics are measured using proxies or less detailed response options. Development of the survey for this protocol aims to (1) focus on tracking social and behavioural risks for poor mental health, (2) incorporate more validated scales and questionnaires to allow for national and international comparison, (3) extend the survey to a sample representative of 8–18 year olds in England and (4) increase pupil involvement to inform research and interventions.

### Population

The target population for the OxWell survey is children and adolescents aged 8–18 in England, and to reach this group the survey recruits school pupils in years 4–13 at state-maintained and independent schools and Further Education Colleges (FECs) in several local authorities across England. The survey aims to attain a diverse and representative school population sample by three means: first, by recruiting pupils via schools in a range of socioeconomic deprivation areas; second, by using an opt-out model with parents to ensure maximum participation; and thirdly, by not trying to identify pupils to encourage accurate responses. To assess diversity, the survey includes immigration status, three categories for gender identity and proxies for socio-economic status. To assess representativeness, analyses will compare the survey sample to school level information made available by the UK government, such as deprivation indices of the school location and the proportion accessing free school meals.^19^ In 2019, a pilot survey recruited pupils from specific year groups in Oxfordshire and in 2020 this increased to 11 local authorities in the counties of Oxfordshire, Berkshire, Buckinghamshire, Gloucestershire and Wiltshire. The number of schools and counties participating will be expanded to further regions including Merseyside for 2021, and we aim to continue to increase participation nationally in subsequent surveys.

### Patient and public involvement

Extensive discussions with school staff and feedback from pupils, parents and other stakeholders from previous versions of the survey have informed development of the survey. For example, questions about mental healthcare in schools and attitudes to allowing linkage of survey data to other databases for research were developed with stakeholders (some of whom might have been service users). After the survey, summaries of results are distributed to all participating schools, for school staff and pupils. The OxWell Study Team works closely with schools, public health teams, clinical commissioning groups and Child and Adolescent Mental Health Services (CAMHS) in selecting the survey content, and our full public and stakeholder activity is detailed in online supplemental appendix 1.

10.1136/bmjopen-2021-052717.supp1 Supplementary data

### Procedures

All primary schools, secondary schools and FECs in participating counties are invited via their local authority to sign up using an online form, and to watch a webinar about the survey and its potential value. Schools that sign up receive a study information pack from the research team. Schools inform parents of the survey and provide parents with instructions on how to opt-out their child. During a designated school period, schools provide survey information (a 3 min video) and login details to pupils who have not been opted-out. Pupils happy to take part then log in, read the instructions, provide online assent (or consent for those aged over 16) and complete the survey. To mitigate difficulties for younger pupils with the online assent process, pupils and school staff are informed about the assent procedure, the assent questions are presented one at a time, and pupils are instructed to ask their teacher if they do not understand a question. The entire session takes pupils 15–40 min, depending on the survey version (divided into three age-appropriate versions) and responses to ‘gateway’ questions that might probe participants with further questions.

### Baseline data collections and planned data collections

Baseline 2019 data collected in-schools: In 2019, we recruited 36 out of a potential 383 Oxfordshire schools (mostly state-maintained and some independent) and collected data from 4290 pupils in UK years 4, 5, 6, 8, 10 and 12 between May and July 2019. Pupils completed the survey in the classroom. Our estimated response rate based on school capacity data was 85% (approx. 5054 were invited by their school). The pupils who did not participate include those who were absent from school on the day of collection, as well as parental opt-outs and pupil opt-outs (records of which were held by schools).

Baseline 2020 data collected during partial school closures: In 2020, access to the survey was adapted for the first UK COVID-19 national lockdown, so that pupils in all year groups (years 4–13) were invited to complete the survey from home or school. While it was not possible to ensure pupils’ complete privacy in the home environment, schools were asked to send survey information and login details directly to pupils via email or the school learning platform, giving them one or more days to complete the survey. The instructions stressed that pupils could skip any questions and the importance of closing the browser window when leaving the survey. Additional questions were included to understand pupils’ experience of the pandemic and school closures. Local authorities and CAMHS teams in Oxfordshire, Berkshire, Wiltshire, Gloucestershire, South Gloucestershire and Buckinghamshire, and six additional schools in Bristol and North Somerset collaborated on the survey. 22 336 pupils logged in to the survey from 238 schools and FECs in June and July 2020, with an estimated response rate of 20% of invited pupils.

Planned 2021 and future data to be collected in-school: The 2021 survey is scheduled for mid-May to July 2021. Pupils from year groups 5–13 will be invited to complete the survey during a dedicated lesson period. Local authorities and CAMHS teams in at least Oxfordshire, Berkshire, Buckinghamshire and Liverpool will lead recruitment for the survey. The plan is to collaborate with the same and more local authorities in subsequent years.

### Measures

The OxWell Student Survey is being tailored to understand the relationship between social and behavioural risk factors, mental health outcomes and care seeking in a school-aged population. Questions address a variety of contextual and lifestyle factors, track in-school interventions and assess attitudes to sensitive data and data linkage, while minimising the collection of personal data. There are three age-appropriate survey versions, for school years 4–7, 8–11, and 12–13 (including FECs). The categories of questions asked in the survey are described in relation to each survey year and age-matched version in table 1. While many measures of mental health and well-being will be retained for comparison with baseline and potentially older surveys, collaborations and stakeholders will inform continued development. Variable guides (online supplemental appendices 2a–c) and other information can be found on the Open Science Framework (https://osf.io/sekhr/).

10.1136/bmjopen-2021-052717.supp2 Supplementary data

10.1136/bmjopen-2021-052717.supp3 Supplementary data

10.1136/bmjopen-2021-052717.supp4 Supplementary data

**Table 1:** Overview of survey questions asked by calendar year and school age group

Category	Question types	Year and age group asked		
		2019	2020	2021*
Demographics	School	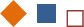	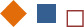	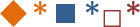
	Gender	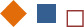	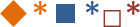	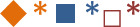
	Age/year group	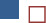	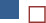	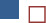
	Born in UK/parents born in UK?	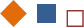	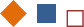	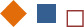
	Living circumstances	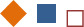	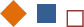	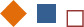
Deprivation	Eligibility for free school meals	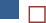	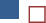	
	Food poverty	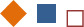	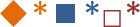	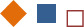
	Parental home ownership			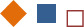
Diet	Eat Breakfast/snacks/ water	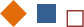	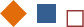	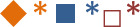
Exercise	No sessions/no hours	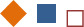	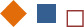	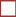
	In school/outside school	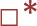	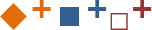	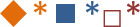
	Barriers/motivation	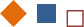	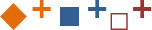	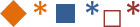
Sleep	Time to bed/time wake up	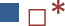	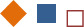	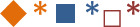
	How long to fall asleep/wake in the night?	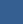	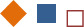	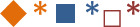
	What before sleep?	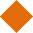	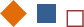	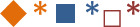
Vulnerability	Feel safe at home/school	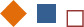	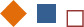	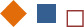
	Bullying (how often, etc)	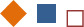	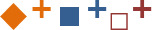	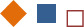
	Child abuse and neglect	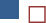	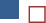	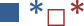
	ACEs	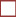	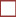	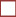
Substance misuse	Smoking	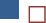	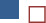	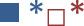
	Alcohol	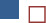	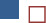	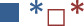
	Illegal and legal drugs	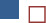	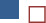	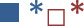
Criminal behaviour	Violence and use of weapons			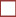
	Gambling	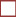	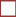	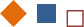
	Online gambling		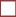	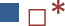
Self-harm	Self-harm	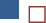	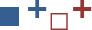	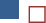
	Self-harm related care seeking	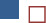	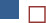	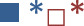
	Familial suicide	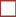	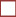	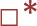
Relationships	Friendships	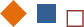	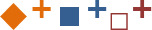	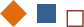
	Abusive relationships	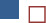	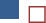	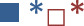
	Loneliness	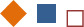	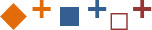	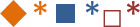
Sexual health	Sexual relationships	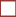	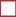	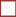
	Access to contraception if needed	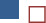	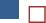	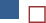
School experience	Exclusion	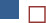	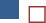	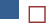
	Attitudes/behaviour at school	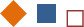	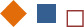	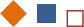
	Pupil agency and empowerment			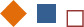
Mental health support	Received support	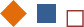	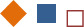	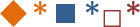
	Would benefit from support	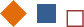	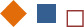	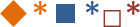
	Barriers/access to support	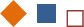	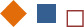	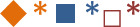
Mental health	RCADS short version		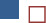	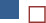
	Bird questionnaire of paranoia			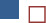
Mental well-being	WEMWBS	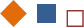	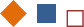	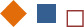
	Life satisfaction	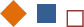	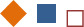	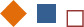
Gaming and social media use	Time spent gaming and on social media			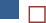
	Gaming addiction			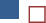
COVID	COVID infection		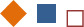	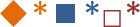
	Vaccine hesitancy			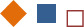
Memory	Ability to concentrate and memory			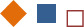
Research	Attitude to providing linkable data	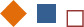	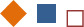	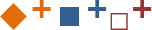
	Did you take part last year?		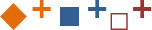	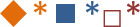

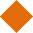
Years 4–7, 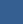
Years 8–11, 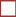
Years 12–13.

+Adapted for COVID pandemic school closures.

*Modified from previous year.

*2021 data collection excludes year 4.

ACEs, Adverse Childhood Experiences; RCADS, Revised Children’s Anxiety and Depression Scales; WEMWBS, Warwick-Edinburgh Mental Well-being Score.

Survey measures in 2019 and 2020: The 2019 and 2020 OxWell surveys used many of the questions from the previous local authority-administered surveys to allow comparison with earlier data collections. All surveys (for all age groups) included the Warwick-Edinburgh Mental Well-being Score (WEMWBS).^20^ Other items measured general well-being, key demographics and a range of school, contextual and behavioural factors (see table 1). Additional questions in the surveys for older pupils (years 8–13 and FECs) included some risk-taking behaviours, past mental health support, one question about witnessing domestic abuse, and number of Adverse Childhood Experiences (ACEs; asked of years 12 and 13 and FECs only).^21^ The 2020 survey included the 25-item version of the Revised Children’s Anxiety and Depression Scales (RCADS)^22 23^ and other questions were adapted to take into account the COVID-19 pandemic. For example, we asked whether pupils thought that anyone in their family had been infected with COVID-19, their worries related to the pandemic, and how the lockdown had affected their home and school lives.^24^

Survey measures in 2021: The 2021 OxWell survey makes a substantial departure from previous surveys. It retains the broad range of topics covered in 2019 and 2020, but substitutes many of the previous questions for those with more in-depth examination of the following topics: substance misuse, child maltreatment, sleep, mental health disorders and experience of a range of mental health support (see table 1). It retains the WEMWBS, RCADS, ACEs, exercise and demographic questions from the previous surveys for comparability. It includes additional questions on pupil agency and empowerment, internet gaming behaviours, sleep patterns, cognition, antisocial behaviour, paranoia-like experiences and vaccine hesitancy.

## Data analysis plan

Here, we provide only an outline of the current analysis plan, but this is expected to develop substantially. This analysis overview focuses on five areas: assessing prevalence of mental health problems; exploring access and attitudes to mental health support; investigating risk and protective factors for poor mental health and well-being; comparing measures of adolescent mental health and well-being; and assessing attitudes to sensitive data and data linkage. Details such as exclusion of survey responses and dealing with missing data will vary depending on the individual research question and analysis, and as such are not reported in this protocol.

### Assessing prevalence of poor mental health in school-aged children and young people

There are a number of advantages for assessing prevalence in the OxWell survey. First, OxWell uses an opt-out approach with parents. A challenge when estimating prevalence is the representativeness of the analysis sample and surveys often miss vulnerable groups who are less likely to respond when explicit (opt-in) consent from parents is required.^25 26^ Second, OxWell collects validated measures of mental well-being, anxiety and depression, and a wide range of experiences and behaviours related to poor mental health. Thirdly, as a repeated cross-sectional survey it can assess change in prevalence (controlling for sample differences), in comparison to single cross-sectional prevalence surveys.

The research questions that will be addressed are:

- What is the prevalence of low mental well-being, depression and anxiety among school pupils in England?
- What is the prevalence of experiences/behaviours associated with poor mental health among adolescents (difficulty sleeping due to worry, generally unhappy, feeling unsafe, lonely, unsatisfied with life, self-harm)?
- What is the impact of environmental and societal changes on prevalence of these mental health-related factors and on the relationships between factors?

Analyses investigating prevalence will take into account differences in the populations accessed by comparing demographics to the census data, and non-response weights can be developed and included in (sensitivity) analyses. We will also model complex interactions between risk and protective factors on mental well-being outcomes and test, for instance, whether the impact of these factors differed before, during, and after pandemic-related school closures.

### Exploring access and attitudes to mental health support

Many young people with mental health difficulties do not access mental healthcare and support.^27^ Understanding the extent to which those not accessing care might want or benefit from mental health support is critical for ensuring mental health provision in schools is acceptable and accessible to young people. The survey asks about receipt of mental health support, desire to access support, and barriers to access. We will explore the following questions:

- What proportion of school pupils have accessed mental health support or services (through their schools, the National Health Service/CAMHS, third sector or remote providers)?
- What proportion of pupils believe they could have benefited from (an alternative source of) support?
- What are the characteristics of pupils who feel they need support but are not accessing it, and how does their mental health compare to the mental health of those who
  1. Have limited experience of accessing support or did not feel the need for it?
  2. Accessed support in the past.
  3. Are currently accessing support?
- For those who feel they could have benefitted from additional support, what are the reasons they give for not accessing it?

There is an increasing policy focus on the role of schools in promoting and protecting young people’s mental health. Recent UK policy changes have introduced Educational Mental Health Practitioners as part of School Mental Health Teams into pilot ‘trailblazer’ schools.^28^ These schools can be identified in the survey and responses by pupils assessed with regard to attitudes and access to services. This provides an opportunity to equip schools and services with relevant results so as to better evaluate current provision. Findings from the OxWell survey will help ensure pupil preferences and responses are summarised for commissioners and service providers.

### Investigating risk and protective factors for poor mental health

An important goal of the survey is to identify protective factors for poor mental health that can be maximised via school interventions. The broad sample and scope of mental health-related measures in the survey provide a unique opportunity to explore interactions between risk and protective factors.

The primary research questions that can be addressed are:

- Which behaviours and support factors are associated with positive mental health and well-being, taking into account demographic and contextual differences and pre-existing vulnerabilities?
- Which risk and contextual factors moderate the benefits of these behaviours and support factors—that is, which pupils are more likely to benefit?

For example, mixed effects analyses will be used to investigate the role risk and protective factors have on mental well-being, depression and anxiety, while controlling for key background characteristics such as age, gender, and socio-demographic deprivation factors, as well as effects reflecting school and year group levels. Similarly, we will explore and the association between paranoid thoughts, poor mental health and access to care seeking. Regression models will be used to investigate factors associated with self-harm. Specific hypotheses will be tested using moderation and mediation analyses with risk and protective factors, such as the extent to which lifestyle factors like exercise might reduce the negative impact of ACEs on mental health and well-being.

### Comparing measures of mental health and well-being

Screening for mental illness in schools is a potential means to preventing long-term negative impacts on multiple outcomes, by offering support or running tailored interventions within schools.^29 30^ Many validated measures of mental illness are designed to be completed by parents or teachers, with self-report versions suitable for adolescents.^26^ At least half of the items are negative statements in the first person, to which participants are asked the extent to which they agree. These negatively worded scales are effective at detecting risk of mental illness, but it is unclear which measures are suitable and acceptable for screening mostly healthy populations.

The survey includes the WEMWBS, a positively worded measure of well-being, asking respondents the extent to which they agree with sentences such as, ‘I’ve been feeling good about the future’.^20^ The WEMWBS is validated as a measure of mental well-being in adolescents of 11 years and older,^31^ but is not a measure of mental illness. One study compared the WEMWBS with validated measures of mental health in adults the General Health Questionnaire, (GHQ-12), and the Health Survey for England, EQ-5D) and found them to have overlapping constructs.^32^ Other measures of well-being have been shown to be distinct from mental illness in 11-year-old children, using the parent-report version of the Strengths and Difficulties Questionnaire.^33^ To our knowledge, no comparison between WEMWBS and self-report measures of mental illness has been reported in adolescents. To understand how well-being and lifestyle factors relate to diagnosable mental health conditions, the survey versions for pupils aged 12 years and older also include the RCADS.^23^

The main research question that can be addressed is:

- To what extent does the WEMWBS well-being score predict diagnosable depression and anxiety in school-aged adolescents?

Analyses will assess the extent to which the mental well-being score, in combination with other items (eg, sleep quality and stress measures), predicts depression and anxiety measured by RCADS. Additional analyses can be used to assess the extent to which well-being predicts which adolescents have accessed or felt they needed mental health support, to assess whether the WEMWBS can inform unmet needs. Pupils in years 4–7 do not complete RCADS, but associations between RCADS, WEMWBS and other measures (eg, disturbed sleep and general happiness) in older pupils could inform the development of population-level mental health measures in younger pupils.

### Assessing attitudes to providing data for research

Considering the opportunities of linking research data to administrative data,^34 35^ pupils’ attitudes to anonymous vs pseudonymous (linkable) research data on the ‘sensitive’ topic of mental health and vulnerability is assessed, and more specifically the extent to which pupils would answer differently if their responses could be linked to their school records. To our knowledge, this is the first large-scale survey to ask children and adolescents their opinions about data linkage. Such information is relevant not only to involve them in the research process, but also to understand which risk factors are difficult to measure in identifiable or linkable form. At the end of the survey, after pupils have provided information of varying sensitivity, a description of the nature and purpose of data linkage for research is presented, drafted based on discussions with a YPAG. Pupils are then asked the extent to which they would mind or answer differently if in future surveys their responses could be linked to other information on them (eg, school or health records).

The primary research questions that can be addressed are:

- Which information are young people most reluctant to provide accurately if their responses could be linked to other information on them?
- Which risk factors relevant to adolescent mental health are most difficult to investigate with linkable (pseudonymous) data?

Analyses incorporating the most sensitive questions on social and behavioural risk factors measured in the survey will be used to answer these research questions. We will seek to validate these measures by comparing responses in the survey to proportions and characteristics of children and adolescents who have consented to data linkage in other studies.

## Ethics and dissemination

### Ethics

The study was reviewed and approved by the University of Oxford Research Ethics Committee (reference R62366). The research team provide all schools that sign up with detailed ethical procedures for the study, information for parents/guardians and pupils, and school-specific login IDs and passwords for their pupils. Schools inform parents 1 week before giving survey and login information to pupils, with contact details for the research team, and instructions on how to opt-out by contacting the school. Schools keep their own records of opt-outs from parents and are instructed not to provide login details to these pupils. Pupil participants are informed about the survey and invited to watch a 3 min video. Participants are asked to give online active assent before completing the survey, or full consent in the case of adolescents over 16 years of age. To protect their privacy, pupils are not asked to provide names, addresses, date of birth or ethnicity; no unique logins are used and IP addresses are not retained. To further ensure confidentiality when completing the survey online, the survey provider is under contract to keep the data safe and to use encryption when transferring the data to the research team, and participants are instructed and reminded to close their browser window when they are finished. Pupils are informed and frequently reminded during the survey that they can stop at any time, as well as leave questions blank and move on to the next question if they do not wish to answer. At the end of the survey pupils are advised as to what they could do if they feel worried or upset about anything, including seeking support from trusted school staff. Information on appropriate online resources supporting adolescent mental health is also provided. In the event that a survey response raises concern about the safety of a participant, the lack of identifiers in the survey means that we are unable to positively identify pupils in order to provide direct referral to appropriate support. However, schools will be notified of the concern, together with the year group and gender of any pupils considered to be at risk.

### Dissemination statement

For the dissemination of results, participating schools and local authorities will be prioritised to receive summaries of findings from their own pupils’ responses within 3 months of data collection, to inform provision of support, services and school interventions directly. Findings will also be shared on social media, via channels directed at children and adolescents. Analyses of the complete data, especially those addressing pressing public health questions, will be presented at scientific meetings with researchers and service providers, and will be submitted for publication in peer-reviewed journals. The data collections will be made available in anonymous form for researchers to apply to analyse, following preparation of data dictionaries and other technical information on the data. A review process will be designed to ensure sound data governance, that research questions fall under the remit of the intended purposes, and to prevent duplication of analyses.

## Discussion

There is increasing attention by policy-makers,^28 36^ research funding bodies^37^ and the general public placed on the mental health of children and adolescents, and the implications that poor mental health in these early years may have on later life, with primary prevention a key strategic target.^38 39^ Yet there are limited current data from diverse and representative samples on current social and behavioural risk factors that contribute to poor mental health in children and adolescents, and on whether the current mental health provision is acceptable.^40 41^ The OxWell survey provides an opportunity to explore these issues in a broad, repeated cross-sectional survey in a large population of school pupils in England. The longer-term goals of this survey are to establish a current evidence-base and foundation for the development and evaluation of mental health interventions in schools, aimed at both prevention of poor mental health and promotion of pupils’ well-being. Utilising input from school staff and mental health service providers, the survey could help to quantify the prevalence of changing risk factors and potential targets for local or national interventions.

There are a number of strengths and limitations to the survey. It provides a diverse sample, which is achieved by recruiting pupils via schools in areas with different socioeconomic deprivation levels, using an opt-out approach with parents, and protecting the identity of the participants to gain their trust. The survey has widespread support from schools and local authorities, which aids not only recruitment of a sample that is larger and more diverse than many samples, but also means that questions can be included that are relevant to all stakeholders and informative to the development of interventions at the community level. There is a limitation in that analyses of repeated cross-sectional data provide very little insight into causality. Differences in data collection methods and non-response biases may also influence representativeness. For example, school signup rates are higher in some local authorities than others, depending on the nature of their contact with schools, which can lead to sampling bias. Notably, the sample gathered in 2020 during the national lockdown may be more biased than the sample collected in 2019 in the classroom setting due to variable school and pupil engagement and issues around access to equipment needed to complete the survey. It is also not possible to ensure privacy for respondents in their home environment, implying that some might have skipped sensitive questions or not been truthful in their responses if they were unable to find time alone. In order to maximise the capabilities of the data and to derive estimates of incidence, measures have been taken to facilitate comparison between past surveys and future data collections. This includes collection of school IDs and year groups, and asking participants whether they completed the survey in the previous year, as well as comparison with school level summaries and weighting of responses to control for representativeness. Therefore, in analyses comparing OxWell data collections, trajectories of school classes can potentially be tracked in a similar way to how individuals’ trajectories can be tracked in longitudinal data. This could facilitate the tailoring of interventions to school classes (rather than to individuals).

Inherent to the limitations of not collecting explicit identifiers, the existing data cannot be linked at the individual level to other outcomes or information. Longitudinal child and adolescent mental health data that can be linked at the individual level, ideally incorporating data from education, health and social care settings, can provide a more comprehensive picture of the factors that influence mental illness, making it possible to follow up participants on multiple outcomes.^35^ However, future linkage to relevant aggregate information is possible at the school or regional level, such as school level deprivation indices or data on school policies, in order to take into account the representativeness of the sample and impact of policy and practice.^42 43^ Furthermore, surveys that do not collect explicit identifiers have their own strengths, as participants might be more honest in their responses to sensitive questions. This study and others could help to inform an approach for investigating risk factors for poor mental health that utilises both pseudonymous and anonymous data.
