# Supplementary material for: Study protocol: the OxWell school survey investigating social, emotional and behavioural factors associated with mental health and well-being
Source: BMJ Open. 2021 Nov 30;11(12):e052717. doi: 10.1136/bmjopen-2021-052717 (PMC9066348; doi:10.1136/bmjopen-2021-052717)
Supplement: Supplementary data [file bmjopen-2021-052717supp001.pdf]

OxWell Study Protocol APPENDIX 1: Public and Stakeholder Involvement

Development of the OxWell survey incorporates substantial input from multiple stakeholders:

- The overarching aim of the survey is to gather direct responses from pupils to help understand how best to support them
- Comparable surveys collected by 3 local authorities in Southern England since 2006 have collected data from over 100,000 pupils, with growing engagement from schools and pupils.
- Since 2019, the research team has been working closely with schools, public health teams, clinical commissioning groups and child and adolescent mental health services in the development of the OxWell survey:
  - A Young Persons Advisory Group was consulted to inform survey questions on allowing linkage of survey data to other databases for research, and on provision of mental health support in schools. Additional input from a Professional Youth Advisor working at the Department of Psychiatry, University of Oxford on the overall structure, graphics and sensitive questions in the survey and other youth working on the dissemination of the survey were incorporated into the 2021 iteration.
  - Pupils' responses to these questions can be used to inform development of the research (including governance aspects), local mental health support and other interventions
  - Clinical service commissioners have helped to formulate the questions around access to mental health services, an area where services are undergoing significant change.
  - School headteachers have helped to identify the areas of greatest concern for their pupils' mental health and to ensure that questions regarding access to mental health support were relevant and meaningful.
  - Feedback from teachers and parents are collated to shape the content and approach of the survey, such as the suitability of questions for different age groups and transparency of the wording in the parent information.
  - Results were presented at a Head Teachers conference, at school INSET days and school governor meetings, from which areas of emerging interest and need were raised to be included in subsequent surveys.
  - A community police officer advised on the appropriateness of the substance misuse and risk questions being asked
  - Child and adolescent researchers involved in the ONS Survey and other large scale studies advised on validated and other measures
- After each data collection, the team prioritises providing schools and local authorities with summaries of results from their own pupils, to guide in-school interventions and local mental health provision.
  - All participating schools are provided with tailored summaries of findings for both school staff and pupils, with access to an online data summary tool for school staff, pupils and parents to analyse some of the data collected.
  - The research team holds webinars with schools and local authorities at every stage of the project, providing tailored information on running the survey, highlighting results, and giving guidance on how to access further information from the online data summary tool.
- A Young Person's Advisory Group (YPAG) will be regularly consulted as the survey develops.
- Two school leavers were employed to develop a range of age-appropriate dissemination materials for the school pupils participating in the survey. This included posters and videos to be shown in school assemblies or classrooms; with an additional 2021 social media dissemination plan.

Mansfield KL, *et al. BMJ Open* 2021; 11:e052717. doi: 10.1136/bmjopen-2021-052717
