# Supplementary material for: Study protocol: the OxWell school survey investigating social, emotional and behavioural factors associated with mental health and well-being
Source: BMJ Open. 2021 Nov 30;11(12):e052717. doi: 10.1136/bmjopen-2021-052717 (PMC9066348; doi:10.1136/bmjopen-2021-052717)
Supplement: Supplementary data [file bmjopen-2021-052717supp002.pdf]

# OxWell VARIABLE GUIDE 2021

## *Questionnaire: Primary (Years 4-7)*

**For the most up-to-date versions, please see https://osf.io/sekhr/**

## Contents

### Variable Guide Table of Contents

**DEMOGRAPHICS** .......... 2
**DIET AND FOOD POVERTY** .......... 3
**SLEEP** .......... 4
**EXERCISE** .......... 7
**SCHOOL EXPERIENCE** .......... 10
**SAFETY** .......... 14
**INTERNET** .......... 15
**COVID** .......... 16
**BULLYING** .......... 17
**MENTAL HEALTH** .......... 19
**MENTAL HEALTH SERVICES** .......... 23
**RELATIONSHIPS** .......... 24
**RESEARCH** .......... 25

# OxWell 2012 Primary

## DEMOGRAPHICS

| CODE | Contingent | Question | Label | Value | Type | Matched | Year Included |
|---|---|---|---|---|---|---|---|
| | | ***Please enter your Year Group*** | *Year group* | 4 - 6 | Numeric | Prim; Sec; FE | 2019, 2020, 2021 |
| | | ***Are you a boy or a girl?*** | *Girl*<br>*Boy*<br>*Prefer not to answer* | F<br>M<br>R | String | Prim; Sec*; FE* | 2019, 2020, 2021* |
| | | ***How old are you*** | *Age in full years* | 8 - 12 | Numeric | Prim; Sec; FE | 2019, 2020, 2021 |
| | | ***Who do you live with?*** | *I live with both of my parents*<br>*I live with one of my parents*<br>*My parents are separated and I live in two homes*<br>*I live with other relatives or adoptive parents*<br>*I live with other people (foster carers, foster home, childrens home)*<br>*I live in more than one place*<br>*I live somewhere else*<br>*I would rather not say* | PARENTS<br>PARENT<br>SEPARATED PARENT<br>RELATIVE<br>OTHERHOME<br>MANY<br>OTHER<br>RF | String | Prim; Sec; FE | 2019*; 2020;2021 |
| | | ***Is your home rented?*** | *Yes*<br>*No*<br>*Don't know* | Y<br>N<br>R | String | Prim; Sec; FE | 2021 |
| | | ***Were you born in the UK*** | *Yes*<br>*No*<br>*Would rather not say* | Y<br>N<br>R | String | Prim; Sec; FE | 2019*;2020; 2021 |
| | | ***Were both of your parents born in the UK?*** | *Yes*<br>*No*<br>*Would rather not say* | Y<br>N<br>R | String | Prim; Sec; FE | 2019*;2020; 2021 |



Note: A * in the *Matched* or *Year Included* columns indicates that the *Question* or *Label* column contain differences in the * indicated year or survey version

OxWell 2012 Primary

## DIET AND FOOD POVERTY

| CODE | Contingent | Question | Label | Value | Type | Matched | Year Included |
|---|---|---|---|---|---|---|---|
| | | ***Do you usually eat breakfast?*** | *Never*<br>*Not often*<br>*Sometimes*<br>*Usually*<br>*Every morning* | 0-12.5<br>12.6-37.5<br>37.6-62.4<br>62.5-87.4<br>87.5-100 | Sliding scale | Prim; Sec; FE | 2019; 2020; 2021 |
| | | ***Some young people go to school or to bed hungry because there is not enough food at home. How often does this happen to you?*** | *Not at all*<br>*Once or twice*<br>*Sometimes*<br>*Most days*<br>*Every day* | 0-12.5<br>12.6-37.5<br>37.6-62.4<br>62.5-87.4<br>87.5-100 | Sliding scale | Prim; Sec; FE | 2019; 2020; 2021 |



Note: A * in the *Matched* or *Year Included* columns indicates that the *Question* or *Label* column contain differences in the * indicated year or survey version

# OxWell 2012 Primary

## SLEEP

| CODE | Contingent | Question | Label | Value | Type | Matched | Year Included |
|---|---|---|---|---|---|---|---|
| | | ***What time (to the nearest hour) did you go to bed last night?*** | *Time to bed (in hour)* | 6pm – 2am or later | String | Prim; Sec; FE | 2019; 2020 |
| | | ***How long do you usually take to fall asleep?*** | *0 mins* | 0-12.5 | Sliding scale | Prim; Sec; FE | 2021 |
| | | | *30 mins (half an hour)* | 12.6-37.5 | | | |
| | | | *60 mins (1 hour)* | 37.6-62.4 | | | |
| | | | *90 mins (1.5 hours)* | 62.5-87.4 | | | |
| | | | *120+ mins (2 hours or more)* | 87.5-100 | | | |
| | | ***What time do you usually go to bed on a school night?*** | *6pm* | 0-12.5 | Sliding scale | Prim; Sec; FE | 2021 |
| | | | *8pm* | 12.6-37.5 | | | |
| | | | *10pm* | 37.6-62.4 | | | |
| | | | *12 midnight* | 62.5-87.4 | | | |
| | | | *2am or later* | 87.5-100 | | | |
| | | ***What time do you usually try to fall asleep on a school night?*** | *6pm* | 0-12.5 | Sliding scale | Prim; Sec; FE | 2021 |
| | | | *8pm* | 12.6-37.5 | | | |
| | | | *10pm* | 37.6-62.4 | | | |
| | | | *12 midnight* | 62.5-87.4 | | | |
| | | | *2am or later* | 87.5-100 | | | |
| | | ***What time do you usually wake up on a school day?*** | *5am* | 0-12.5 | Sliding scale | Prim; Sec; FE | 2021 |
| | | | *7am* | 12.6-37.5 | | | |
| | | | *9am* | 37.6-62.4 | | | |
| | | | *11am* | 62.5-87.4 | | | |
| | | | *1pm or later* | 87.5-100 | | | |
| | | ***What time do you usually go to bed at the weekend?*** | *6pm* | 0-12.5 | Sliding scale | Prim; Sec; FE | 2021 |
| | | | *8pm* | 12.6-37.5 | | | |
| | | | *10pm* | 37.6-62.4 | | | |
| | | | *12 midnight* | 62.5-87.4 | | | |
| | | | *2am or later* | 87.5-100 | | | |
| | | ***What time do you usually try to fall asleep at the weekend?*** | *6pm* | 0-12.5 | Sliding scale | Prim; Sec; FE | 2021 |
| | | | *8pm* | 12.6-37.5 | | | |
| | | | *10pm* | 37.6-62.4 | | | |
| | | | *12 midnight* | 62.5-87.4 | | | |
| | | | *2am or later* | 87.5-100 | | | |



Note: A * in the *Matched* or *Year Included* columns indicates that the *Question* or *Label* column contain differences in the * indicated year or survey version

OxWell 2012 Primary

| | | | | | | |
|---|---|---|---|---|---|---|
| | ***What time do you usually wake up at the weekend?*** | *5am*<br>*7am*<br>*9am*<br>*11am*<br>*1pm or later* | 0-12.5<br>12.6-37.5<br>37.6-62.4<br>62.5-87.4<br>87.5-100 | Sliding scale | Prim; Sec; FE | 2021 |
| | ***People sometimes feel sleepy during the daytime. During your daytime activities, how much of a problem do you have with sleepiness (feeling sleepy, struggling to stay awake)?*** | *No problem at all*<br>*A little problem*<br>*More than a little problem*<br>*A big problem*<br>*A very big problem* | 0-12.5<br>12.6-37.5<br>37.6-62.4<br>62.5-87.4<br>87.5-100 | Sliding scale | Prim; Sec; FE | 2021 |
| | ***Thinking about the past month, to what extent has poor sleep troubled you in general?*** | *Not at all*<br>*A little*<br>*Somewhat*<br>*Much*<br>*Very much* | NOTATALL<br>LITTLE<br>SOMEWHAT<br>MUCH<br>VERYMUCH | String | Prim; Sec; FE | 2021 |
| | ***Thinking about a typical night in the last month, how many nights a week do you have a problem with your sleep?*** | *0-1*<br>*2*<br>*3*<br>*4*<br>*5-7* | 0TO1<br>2<br>3<br>4<br>5TO7 | String | Prim; Sec; FE | 2021 |
| | ***On a school night (when you have lessons the next day), who usually sets your bedtime?*** | *Yourself*<br>*Parent/carer/guardian/*<br>*other family member*<br>*other* | SELF<br>CARER<br>##<br>OTHER | String | Prim; Sec; FE | 2021 |
| | ***On a weekend night (no lessons the next day), who usually sets your bedtime?*** | *Yourself*<br>*Parent/carer/guardian/*<br>*other family member*<br>*other* | SELF<br>CARER<br>##<br>OTHER | String | Prim; Sec; FE | 2021 |
| | ***On school nights (when you have lessons the next day), do you have a rule or set time in your house about when you are supposed to turn off or put away computers, phones or other electronics?*** | *Yes*<br>*No* | Y<br>N | String | Prim; Sec; FE | 2021 |
| | ***How often do you use social media (e.g. tik-tok, instagram) in the hour before you intend to go to sleep?*** | *Never*<br>*Rarely (1-2 times a month)* | 0-12.5<br>12.6-37.5 | Sliding scale | Prim; Sec; FE | 2021 |



Note: A * in the *Matched* or *Year Included* columns indicates that the *Question* or *Label* column contain differences in the * indicated year or survey version

OxWell 2012 Primary

| | | | | | | |
|---|---|---|---|---|---|---|
| | | *Sometimes (1-2 times a week)* | 37.6-62.4 | | | |
| | | *Often (3-4 times a week)* | 62.5-87.4 | | | |
| | | *Daily* | 87.5-100 | | | |
| | ***How often do you play video games in the hour before you intend to go to sleep (including games on consoles, computer, tablet, mobile phone or other portable gaming device)?*** | *Never* | 0-12.5 | Sliding scale | Prim; Sec; FE | 2021 |
| | | *Rarely (1-2 times a month)* | 12.6-37.5 | | | |
| | | *Sometimes (1-2 times a week)* | 37.6-62.4 | | | |
| | | *Often (3-4 times a week)* | 62.5-87.4 | | | |
| | | *Daily* | 87.5-100 | | | |

Note: A * in the *Matched* or *Year Included* columns indicates that the *Question* or *Label* column contain differences in the * indicated year or survey version

OxWell 2012 Primary

## EXERCISE

| CODE | Contingent | Question | Label | Value | Type | Matched | Year Included |
|---|---|---|---|---|---|---|---|
| | | ***About how many hours physical activity or exercise do you do over a whole week (in and out of school, during term-time)*** | *1 hour or less)* | 0-12.5 | Sliding scale | Prim; Sec; FE | 2020; 2021* |
| | | | *3 hours* | 12.6-37.5 | | | |
| | | | *6 hours* | 37.6-62.4 | | | |
| | | | *9 hours* | 62.5-87.4 | | | |
| | | | *12 hours or more* | 87.5-100 | | | |
| | | ***How many times per week do you normally do PE/sports during school time?*** | *0* | 0-12.5 | Sliding scale | Prim; Sec; FE | 2021 |
| | | | *3* | 12.6-37.5 | | | |
| | | | *6* | 37.6-62.4 | | | |
| | | | *9* | 62.5-87.4 | | | |
| | | | *12 or more* | 87.5-100 | | | |
| | | ***How many times per week do you do sports or exercise outside of school hours?*** | *0* | 0-12.5 | Sliding scale | Prim; Sec; FE | 2021 |
| | | | *3* | 12.6-37.5 | | | |
| | | | *6* | 37.6-62.4 | | | |
| | | | *9* | 62.5-87.4 | | | |
| | | | *12 or more* | 87.5-100 | | | |
| | | ***Is most of your exercise at school or outside of school?*** | *Only at* | 0-12.5 | Sliding scale | Prim; Sec; FE | 2021 |
| | | | *Mostly at* | 12.6-37.5 | | | |
| | | | *Same in/out of* | 37.6-62.4 | | | |
| | | | *Mostly outside* | 62.5-87.4 | | | |
| | | | *All outside* | 87.5-100 | | | |
| | | ***Compared to before the first lockdown, how much time do you spend doing sports or exercise now?*** | *Much less* | 0-12.5 | Sliding scale | Prim; Sec; FE | 2021 |
| | | | *Slightly less* | 12.6-37.5 | | | |
| | | | *The same amount* | 37.6-62.4 | | | |
| | | | *Slightly more* | 62.5-87.4 | | | |
| | | | *Much more* | 87.5-100 | | | |
| | | ***How much do you enjoy physical exercise outside of school time?*** | *Never* | 0-12.5 | Sliding scale | Prim; Sec; FE | 2021 |
| | | | *Not often* | 12.6-37.5 | | | |
| | | | *Sometimes* | 37.6-62.4 | | | |
| | | | *Quite often* | 62.5-87.4 | | | |
| | | | *Always* | 87.5-100 | | | |
| | | ***Would you like to do more sports/exercise?*** | *Yes* | Y | String | Prim; Sec; FE | 2020; 2021 |
| | | | *No* | N | | | |



Note: A * in the *Matched* or *Year Included* columns indicates that the *Question* or *Label* column contain differences in the * indicated year or survey version

OxWell 2012 Primary

| | | | | | | |
|---|---|---|---|---|---|---|
| | ***How much do you normally enjoy your PE lessons?*** | *Never* | 0-12.5 | String | Prim; Sec; FE | 2020; 2021 |
| | | *Not often* | 12.6-37.5 | | | |
| | | *Sometimes* | 37.6-62.4 | | | |
| | | *Quite often* | 62.5-87.4 | | | |
| | | *Always* | 87.5-100 | | | |
| | ***How important are the following in your decision to do sport or physical activity:*** | | | | | |
| | ***Spend time with friends and meet new people*** | *Not at all important* | 0-12.5 | Sliding scale | Prim; Sec; FE | 2019; 2021 |
| | | *Not very important* | 12.6-37.5 | | | |
| | | *Quite important* | 37.6-62.4 | | | |
| | | *Important* | 62.5-87.4 | | | |
| | | *Very important* | 87.5-100 | | | |
| | ***Exercise get fit*** | *Not at all important* | 0-12.5 | Sliding scale | Prim; Sec; FE | 2019; 2020; 2021 |
| | | *Not very important* | 12.6-37.5 | | | |
| | | *Quite important* | 37.6-62.4 | | | |
| | | *Important* | 62.5-87.4 | | | |
| | | *Very important* | 87.5-100 | | | |
| | ***Unwind/reduce stress*** | *Not at all important* | 0-12.5 | Sliding scale | Prim; Sec; FE | 2019; 2020; 2021 |
| | | *Not very important* | 12.6-37.5 | | | |
| | | *Quite important* | 37.6-62.4 | | | |
| | | *Important* | 62.5-87.4 | | | |
| | | *Very important* | 87.5-100 | | | |
| | ***Weight management/body shaping*** | *Not at all important* | 0-12.5 | Sliding scale | Prim; Sec; FE | 2019; 2020; 2021 |
| | | *Not very important* | 12.6-37.5 | | | |
| | | *Quite important* | 37.6-62.4 | | | |
| | | *Important* | 62.5-87.4 | | | |
| | | *Very important* | 87.5-100 | | | |
| | ***Learn a new skill*** | *Not at all important* | 0-12.5 | Sliding scale | Prim; Sec; FE | 2019; 2020; 2021 |
| | | *Not very important* | 12.6-37.5 | | | |
| | | *Quite important* | 37.6-62.4 | | | |
| | | *Important* | 62.5-87.4 | | | |
| | | *Very important* | 87.5-100 | | | |
| | ***Improve performance/do well competitively*** | *Not at all important* | 0-12.5 | Sliding scale | Prim; Sec; FE | 2019; 2020; 2021 |
| | | *Not very important* | 12.6-37.5 | | | |
| | | *Quite important* | 37.6-62.4 | | | |
| | | *Important* | 62.5-87.4 | | | |
| | | *Very important* | 87.5-100 | | | |
| | ***Enjoyment - feeling good physically/mentally*** | *Not at all important* | 0-12.5 | Sliding scale | Prim; Sec; FE | 2019; 2020; 2021 |
| | | *Not very important* | 12.6-37.5 | | | |
| | | *Quite important* | 37.6-62.4 | | | |
| | | *Important* | 62.5-87.4 | | | |



Note: A * in the *Matched* or *Year Included* columns indicates that the *Question* or *Label* column contain differences in the * indicated year or survey version

OxWell 2012 Primary

| | | | | | | |
|---|---|---|---|---|---|---|
| | | *Very important* | 87.5-100 | | | |
| | ***Have to do it for others - school/team/family*** | *Not at all important* | 0-12.5 | Sliding scale | Prim; Sec; FE | 2019; 2020; 2021 |
| | | *Not very important* | 12.6-37.5 | | | |
| | | *Quite important* | 37.6-62.4 | | | |
| | | *Important* | 62.5-87.4 | | | |
| | | *Very important* | 87.5-100 | | | |
| | ***How do you normally (i.e. for most of the journey) travel to school?*** | *I travel by vehicle (e.g. bus, car, van, train, motorbike)* | TRAVELVEHICLE | Sliding scale | Prim; Sec; FE | 2019*; 2021 |
| | | *I do not travel by vehicle (e.g. walk, cycle, skate, skoot, blades)* | TRAVELWALK | | | |



Note: A * in the *Matched* or *Year Included* columns indicates that the *Question* or *Label* column contain differences in the * indicated year or survey version

OxWell 2012 Primary

## SCHOOL EXPERIENCE

| CODE | Contingent | Question | Label | Value | Type | Matched | Year Included |
|---|---|---|---|---|---|---|---|
| | | ***How much help and support with learning do you feel that you get at school?*** | *No help at all* | 0-12.5 | Sliding scale | Prim; Sec; FE | 2019; 2020; 2021 |
| | | | *Not enough help* | 12.6-37.5 | | | |
| | | | *Just about enough help* | 37.6-62.4 | | | |
| | | | *Most of the help I need* | 62.5-87.4 | | | |
| | | | *All of the help I need* | 87.5-100 | | | |
| | | ***How much help and support do you feel you get with your homework from the people you live with?*** | *No help at all* | 0-12.5 | Sliding scale | Prim; Sec; FE | 2019; 2020; 2021 |
| | | | *Not enough help* | 12.6-37.5 | | | |
| | | | *Just about enough help* | 37.6-62.4 | | | |
| | | | *Most of the help I need* | 62.5-87.4 | | | |
| | | | *All of the help I need* | 87.5-100 | | | |
| | | ***How would you describe being at your school?*** | | | | | 2019*; 2021 |
| | | ***I enjoy my school*** | *Strongly Disagree* | 0-12.5 | Sliding scale | Prim; Sec; FE | 2019; 2021 |
| | | | *Disagree* | 12.6-37.5 | | | |
| | | | *Neither Agree nor disagree* | 37.6-62.4 | | | |
| | | | *Agree* | 62.5-87.4 | | | |
| | | | *Strongly Agree* | 87.5-100 | | | |
| | | ***I try my best at school*** | *Strongly Disagree* | 0-12.5 | Sliding scale | Prim; Sec; FE | 2019; 2021 |
| | | | *Disagree* | 12.6-37.5 | | | |
| | | | *Neither Agree nor disagree* | 37.6-62.4 | | | |
| | | | *Agree* | 62.5-87.4 | | | |
| | | | *Strongly Agree* | 87.5-100 | | | |
| | | ***I learn a lot at school*** | *Strongly Disagree* | 0-12.5 | Sliding scale | Prim; Sec; FE | 2019; 2021 |
| | | | *Disagree* | 12.6-37.5 | | | |
| | | | *Neither Agree nor disagree* | 37.6-62.4 | | | |
| | | | *Agree* | 62.5-87.4 | | | |
| | | | *Strongly Agree* | 87.5-100 | | | |
| | | ***My school is giving me useful skills and knowledge*** | *Strongly Disagree* | 0-12.5 | Sliding scale | Prim; Sec; FE | 2019; 2021 |
| | | | *Disagree* | 12.6-37.5 | | | |
| | | | *Neither Agree nor disagree* | 37.6-62.4 | | | |
| | | | *Agree* | 62.5-87.4 | | | |
| | | | *Strongly Agree* | 87.5-100 | | | |
| | | ***My school has lots of activities (like sport and drama) to take part in at lunchtime or after school*** | *Strongly Disagree* | 0-12.5 | Sliding scale | Prim; Sec; FE | 2019; 2021 |
| | | | *Disagree* | 12.6-37.5 | | | |



Note: A * in the *Matched* or *Year Included* columns indicates that the *Question* or *Label* column contain differences in the * indicated year or survey version

OxWell 2012 Primary

| | | | | | | |
|---|---|---|---|---|---|---|
| | | *Neither Agree nor disagree* | 37.6-62.4 | | | |
| | | *Agree* | 62.5-87.4 | | | |
| | | *Strongly Agree* | 87.5-100 | | | |
| | ***My teacher(s) make my lessons fun and interesting*** | *Strongly Disagree* | 0-12.5 | Sliding scale | Prim; Sec; FE* | 2019; 2021 |
| | | *Disagree* | 12.6-37.5 | | | |
| | | *Neither Agree nor disagree* | 37.6-62.4 | | | |
| | | *Agree* | 62.5-87.4 | | | |
| | | *Strongly Agree* | 87.5-100 | | | |
| | ***My teacher(s) tell me how I am doing with my work*** | *Strongly Disagree* | 0-12.5 | Sliding scale | Prim; Sec; FE* | 2019; 2021 |
| | | *Disagree* | 12.6-37.5 | | | |
| | | *Neither Agree nor disagree* | 37.6-62.4 | | | |
| | | *Agree* | 62.5-87.4 | | | |
| | | *Strongly Agree* | 87.5-100 | | | |
| | ***Other pupils often disrupt my lessons*** | *Strongly Disagree* | 0-12.5 | Sliding scale | Prim; Sec; FE | 2019; 2021 |
| | | *Disagree* | 12.6-37.5 | | | |
| | | *Neither Agree nor disagree* | 37.6-62.4 | | | |
| | | *Agree* | 62.5-87.4 | | | |
| | | *Strongly Agree* | 87.5-100 | | | |
| | ***I get enough help at school with learning*** | *Strongly Disagree* | 0-12.5 | Sliding scale | Prim; Sec; FE | 2019; 2021 |
| | | *Disagree* | 12.6-37.5 | | | |
| | | *Neither Agree nor disagree* | 37.6-62.4 | | | |
| | | *Agree* | 62.5-87.4 | | | |
| | | *Strongly Agree* | 87.5-100 | | | |
| | ***I get enough help at school when I am upset*** | *Strongly Disagree* | 0-12.5 | Sliding scale | Prim; Sec*; FE* | 2019; 2021 |
| | | *Disagree* | 12.6-37.5 | | | |
| | | *Neither Agree nor disagree* | 37.6-62.4 | | | |
| | | *Agree* | 62.5-87.4 | | | |
| | | *Strongly Agree* | 87.5-100 | | | |
| | ***I feel stressed by school work*** | *Strongly Disagree* | 0-12.5 | Sliding scale | Prim; Sec; FE | 2019; 2021 |
| | | *Disagree* | 12.6-37.5 | | | |
| | | *Neither Agree nor disagree* | 37.6-62.4 | | | |
| | | *Agree* | 62.5-87.4 | | | |
| | | *Strongly Agree* | 87.5-100 | | | |
| | ***I worry about going to school*** | *Strongly Disagree* | 0-12.5 | Sliding scale | Prim; Sec; FE | 2019; 2021 |
| | | *Disagree* | 12.6-37.5 | | | |
| | | *Neither Agree nor disagree* | 37.6-62.4 | | | |
| | | *Agree* | 62.5-87.4 | | | |



Note: A * in the *Matched* or *Year Included* columns indicates that the *Question* or *Label* column contain differences in the * indicated year or survey version

OxWell 2012 Primary

| | | | | | | |
|---|---|---|---|---|---|---|
| | | *Strongly Agree* | 87.5-100 | | | |
| | ***I am often in trouble*** | *Strongly Disagree* | 0-12.5 | Sliding scale | Prim; Sec; FE | 2019; 2021 |
| | | *Disagree* | 12.6-37.5 | | | |
| | | *Neither Agree nor disagree* | 37.6-62.4 | | | |
| | | *Agree* | 62.5-87.4 | | | |
| | | *Strongly Agree* | 87.5-100 | | | |
| | ***I am often aggressive or violent*** | *Strongly Disagree* | 0-12.5 | Sliding scale | Prim; Sec; FE | 2019; 2021 |
| | | *Disagree* | 12.6-37.5 | | | |
| | | *Neither Agree nor disagree* | 37.6-62.4 | | | |
| | | *Agree* | 62.5-87.4 | | | |
| | | *Strongly Agree* | 87.5-100 | | | |
| | ***I usually achieve top marks at my school*** | *Strongly Disagree* | 0-12.5 | Sliding scale | Prim; Sec; FE | 2019; 2021 |
| | | *Disagree* | 12.6-37.5 | | | |
| | | *Neither Agree nor disagree* | 37.6-62.4 | | | |
| | | *Agree* | 62.5-87.4 | | | |
| | | *Strongly Agree* | 87.5-100 | | | |
| | ***I am happy to use the school toilets*** | *Strongly Disagree* | 0-12.5 | Sliding scale | Prim; Sec; FE | 2019; 2021 |
| | | *Disagree* | 12.6-37.5 | | | |
| | | *Neither Agree nor disagree* | 37.6-62.4 | | | |
| | | *Agree* | 62.5-87.4 | | | |
| | | *Strongly Agree* | 87.5-100 | | | |
| | ***How much do you think the school council and pupils views make a difference at your school?*** | *Not at all* | 0-12.5 | Sliding scale | Prim; Sec | 2019; 2021 |
| | | *Not very* | 12.6-37.5 | | | |
| | | *Neither/Don't Know* | 37.6-62.4 | | | |
| | | *Quite a lot* | 62.5-87.4 | | | |
| | | *Very much* | 87.5-100 | | | |
| | ***About speaking out:*** | | | String | Prim; Sec; FE | 2021 |
| | ***Adults in my school ask for my opinion on things*** | *Never/Rarely* | NEVER | | | |
| | | *Sometimes* | SOMETIMES | | | |
| | | *Often* | OFTEN | | | |
| | ***Adults in my school listen when I share my opinion*** | *Never/Rarely* | NEVER | | | |
| | | *Sometimes* | SOMETIMES | | | |
| | | *Often* | OFTEN | | | |
| | ***My school friends ask my advice when they have a problem*** | *Never/Rarely* | NEVER | | | |
| | | *Sometimes* | SOMETIMES | | | |
| | | *Often* | OFTEN | | | |



Note: A * in the *Matched* or *Year Included* columns indicates that the *Question* or *Label* column contain differences in the * indicated year or survey version

OxWell 2012 Primary

| | | | | | | |
|---|---|---|---|---|---|---|
| | ***If I see something wrong in school I feel I can tell someone and they will listen*** | *Never/Rarely* | NEVER | | | |
| | | *Sometimes* | SOMETIMES | | | |
| | | *Often* | OFTEN | | | |
| | ***I can speak up in class when I have a comment or question*** | *Never/Rarely* | NEVER | | | |
| | | *Sometimes* | SOMETIMES | | | |
| | | *Often* | OFTEN | | | |
| | ***I can speak up when I see someone else being hurt*** | *Never/Rarely* | NEVER | | | |
| | | *Sometimes* | SOMETIMES | | | |
| | | *Often* | OFTEN | | | |
| | ***I can ask adults in my school for help when I need it*** | *Never/Rarely* | NEVER | | | |
| | | *Sometimes* | SOMETIMES | | | |
| | | *Often* | OFTEN | | | |
| | ***How much do you agree with the following statement: I identify with my school community*** | *Fully disagree* | FULLDISAGREE | String | Prim; Sec; FE | 2021 |
| | | *Disagree* | DISAGREE | | | |
| | | *Somewhat disagree* | SOMEDISAGREE | | | |
| | | *Neither agree nor disagree* | NEITHER | | | |
| | | *Somewhat agree* | SOMEAGREE | | | |
| | | *Agree* | AGREE | | | |
| | | *Fully agree* | FULLAGREE | | | |



Note: A * in the *Matched* or *Year Included* columns indicates that the *Question* or *Label* column contain differences in the * indicated year or survey version

# OxWell 2012 Primary

## SAFETY

| CODE | Contingent | Question | Label | Value | Type | Matched | Year Included |
|---|---|---|---|---|---|---|---|
| | | ***When you are at school, how safe do you feel?*** | *Very unsafe* | 0-12.5 | Sliding scale | Prim; Sec; FE | 2019; 2020; 2021 |
| | | | *Unsafe* | 12.6-37.5 | | | |
| | | | *Neither safe nor unsafe* | 37.6-62.4 | | | |
| | | | *Safe* | 62.5-87.4 | | | |
| | | | *Very safe* | 87.5-100 | | | |
| | | ***How safe do you feel at home or the place where you live?*** | *Very unsafe* | 0-12.5 | Sliding scale | Prim; Sec; FE | 2019; 2020; 2021 |
| | | | *Unsafe* | 12.6-37.5 | | | |
| | | | *Neither safe nor unsafe* | 37.6-62.4 | | | |
| | | | *Safe* | 62.5-87.4 | | | |
| | | | *Very safe* | 87.5-100 | | | |
| | | ***How safe do you feel when crossing roads or walking near roads?*** | *Very unsafe* | 0-12.5 | Sliding scale | Prim | 2019; 2021 |
| | | | *Unsafe* | 12.6-37.5 | | | |
| | | | *Neither safe nor unsafe* | 37.6-62.4 | | | |
| | | | *Safe* | 62.5-87.4 | | | |
| | | | *Very safe* | 87.5-100 | | | |
| | | ***How safe do you feel riding your bike on the roads?*** | *Very unsafe* | 0-12.5 | Sliding scale | Prim | 2019; 2021 |
| | | | *Unsafe* | 12.6-37.5 | | | |
| | | | *Neither safe nor unsafe* | 37.6-62.4 | | | |
| | | | *Safe* | 62.5-87.4 | | | |
| | | | *Very safe* | 87.5-100 | | | |



Note: A * in the *Matched* or *Year Included* columns indicates that the *Question* or *Label* column contain differences in the * indicated year or survey version

OxWell 2012 Primary

## INTERNET

| CODE | Contingent | Question | Label | Value | Type | Matched | Year Included |
|---|---|---|---|---|---|---|---|
| | | ***Does an adult usually check what you are doing on the internet?*** | *Yes* | Y | String | Prim | 2019; 2021 |
| | | | *No* | N | | | |
| | | ***Approximately how much time did you spend last night online/playing computer games on your computer, laptop, tablet, mobile phone or games station?*** | *Under 1 hour* | 0-12.5 | Sliding scale | Prim | 2019; 2021 |
| | | | *2 hours* | 12.6-37.5 | | | |
| | | | *4 hours* | 37.6-62.4 | | | |
| | | | *6 hours* | 62.5-87.4 | | | |
| | | | *More than 8 hours* | 87.5-100 | | | |
| | | ***Do you argue with or feel criticised by friends, parents or carers about the amount of time you spend online?*** | *Never* | 0-12.5 | Sliding scale | Prim | 2019; 2021 |
| | | | *Not often* | 12.6-37.5 | | | |
| | | | *Sometimes* | 37.6-62.4 | | | |
| | | | *Quite often* | 62.5-87.4 | | | |
| | | | *Always* | 62.5-87.4 | | | |
| | | ***Have you ever met a person in the real world that you got to know first online?*** | *Yes* | Y | String | Prim; Sec | 2019; 2021 |
| | | | *No* | N | | | |
| | | ***What was the age of the person that you met?*** | *About the same age* | SAME | String | Prim, Sec | 2019; 2021 |
| | | | *Younger* | YOUNGER | | | |
| | | | *Older* | OLDER | | | |
| | | | *An adult* | ADULT | | | |
| | | | *Would rather not say* | R | | | |
| | | ***Did you take someone with you when you met up with them?*** | *I went on my own* | OWN | String | Prim, Sec | 2019; 2021 |
| | | | *I went with a parent/carer* | PARCAR | | | |
| | | | *I went with another adult* | ADULT | | | |
| | | | *I went with my brother/sister* | SIBLING | | | |
| | | | *I went with a friend* | FRIEND | | | |



Note: A * in the *Matched* or *Year Included* columns indicates that the *Question* or *Label* column contain differences in the * indicated year or survey version

OxWell 2012 Primary

## COVID

| CODE | Contingent | Question | Label | Value | Type | Matched | Year Included |
|---|---|---|---|---|---|---|---|
| | | ***Do you think you have had Covid?*** | *No* | N | String | Prim; Sec; FE | 2021 |
| | | | *Possibly* | POSSIBLY | | | |
| | | | *Probably* | PROBABLY | | | |
| | | | *Yes (confirmed by test)* | Y | | | |
| | | ***Compared to before the first lockdown, how easy have you found it to remember things?*** | *Very difficult* | 0-12.5 | Sliding scale | Prim; Sec; FE | 2021 |
| | | | *Quite difficult* | 12.6-37.5 | | | |
| | | | *The same* | 37.6-62.4 | | | |
| | | | *Quite easy* | 62.5-87.4 | | | |
| | | | *Very easy* | 87.5-100 | | | |
| | | ***Compared to before the first lockdown, how easy have you found it to concentrate?*** | *Very difficult* | 0-12.5 | Sliding scale | Prim; Sec; FE | 2021 |
| | | | *Quite difficult* | 12.6-37.5 | | | |
| | | | *The same* | 37.6-62.4 | | | |
| | | | *Quite easy* | 62.5-87.4 | | | |
| | | | *Very easy* | 87.5-100 | | | |
| | | ***Compared to before the first lockdown, how easy have you found it to think clearly?*** | *Very difficult* | 0-12.5 | Sliding scale | Prim; Sec; FE | 2021 |
| | | | *Quite difficult* | 12.6-37.5 | | | |
| | | | *The same* | 37.6-62.4 | | | |
| | | | *Quite easy* | 62.5-87.4 | | | |
| | | | *Very easy* | 87.5-100 | | | |
| | | ***Would you take a COVID-19 vaccine (approved for use in the UK) if offered?*** | *Eager to get a COVID-19 vaccine* | EAGER | String | Prim; Sec; FE | 2021 |
| | | | *Willing to get a COVID-19 vaccine* | WILLING | | | |
| | | | *Not bothered about getting a COVID-19 vaccine* | NOTBOTHERED | | | |
| | | | *Unwilling to get a COVID-19 vaccine* | UNWILLING | | | |
| | | | *Anti-vaccination for COVID-19* | ANTIVACCINATION | | | |
| | | | *Don't know* | DONTKNOW | | | |
| | | | *Don't know what this means* | NOTUNDERSTAND | | | |



Note: A * in the *Matched* or *Year Included* columns indicates that the *Question* or *Label* column contain differences in the * indicated year or survey version

OxWell 2012 Primary

## BULLYING

| CODE | Contingent | Question | Label | Value | Type | Matched | Year Included |
|---|---|---|---|---|---|---|---|
| | | ***How well do you think your school deals with bullying*** | *Very badly* | 0-12.5 | Sliding scale | Prim; Sec; FE | 2019; 2021 |
| | | | *Not very well* | 12.6-37.5 | | | |
| | | | *Average* | 37.6-62.4 | | | |
| | | | *Quite well* | 62.5-87.4 | | | |
| | | | *Extremely well* | 87.5-100 | | | |
| | | ***Have you been bullied in the last year?*** | *No* | 0-12.5 | Sliding scale | Prim*; Sec; FE | 2019*; 2020; 2021 |
| | | | *Not often (e.g. once or twice)* | 12.6-37.5 | | | |
| | | | *Sometimes (e.g. monthly)* | 37.6-62.4 | | | |
| | | | *Quite often (e.g. weekly)* | 62.5-87.4 | | | |
| | | | *Most days* | 87.5-100 | | | |
| | | ***Is the bullying still happening?*** | Yes | Y | String | Prim; Sec; FE | 2019; 2021 |
| | | | No | N | | | |
| | | | Would rather not say | R | | | |
| | | ***If you have been bullied in the last year in what way did it happen?*** | | | Tick box | Prim; Sec; FE | 2019*; 2020; 2021 |
| | | | Physical | Y | | | |
| | | | Verbal | Y | | | |
| | | | Cyber | Y | | | |
| | | | Isolated or Excluded | Y | | | |
| | | | Other | Y | | | |
| | | ***If you have ever been bullied, did someone help you deal with it?*** | | | Tick box | Prim; Sec; FE | 2019; 2021* |
| | | | Parent, step-parent or carer | Y | | | |
| | | | Someone else in your family | Y | | | |
| | | | Friend(s) | Y | | | |
| | | | A teacher or other adult at your school | Y | | | |
| | | | A mental health worker (from outside the school) who visits the school | Y | | | |
| | | | A doctor, nurse (outside school), psychologist | Y | | | |
| | | | A counsellor, youth worker or social worker | Y | | | |
| | | | Internet advice sites, forums, chatrooms etc | Y | | | |
| | | | No-one | Y | | | |



Note: A * in the *Matched* or *Year Included* columns indicates that the *Question* or *Label* column contain differences in the * indicated year or survey version

# OxWell 2012 Primary

| | | | | | | |
|---|---|---|---|---|---|---|
| | ***Where does the bullying usually happen?*** | The journey to/from school | TOFROMSCHOOL | String | Prim; Sec; FE | 2019; 2021* |
| | | Online/social media sites | INTERNET | | | |
| | | During lessons | CLASSROOM | | | |
| | | In school but outside of lessons | OTHERSCHOL | | | |
| | | At home | HOME | | | |
| | | The place you are living now | PLACEMENT | | | |
| | | Outside | OUTSIDE | | | |
| | | Other | OTHER | | | |
| | ***What do pupils in your school do if they see someone being bullied?*** | | | Tick box | | 2021 |
| | | Try to stop the bullying | Y | | | |
| | | Tell a teacher | Y | | | |
| | | Laugh | Y | | | |
| | | Join in | Y | | | |
| | | Nothing | Y | | | |



Note: A * in the *Matched* or *Year Included* columns indicates that the *Question* or *Label* column contain differences in the * indicated year or survey version

OxWell 2012 Primary

## MENTAL HEALTH

| CODE | Contingent | Question | Label | Value | Type | Matched | Year Included |
|---|---|---|---|---|---|---|---|
| | | ***I've been feeling optimistic about the future (optimistic means hopeful and confident)*** | *None of the time* | 0-12.5 | Sliding scale | Prim*; Sec; FE | 2019; 2020; 2021 |
| | | | *Rarely* | 12.6-37.5 | | | |
| | | | *Some of the time* | 37.6-62.4 | | | |
| | | | *Often* | 62.5-87.4 | | | |
| | | | *All of the time* | 87.5-100 | | | |
| | | ***I've been feeling useful*** | *None of the time* | 0-12.5 | Sliding scale | Prim; Sec; FE | 2019; 2020; 2021 |
| | | | *Rarely* | 12.6-37.5 | | | |
| | | | *Some of the time* | 37.6-62.4 | | | |
| | | | *Often* | 62.5-87.4 | | | |
| | | | *All of the time* | 87.5-100 | | | |
| | | ***I've been feeling relaxed*** | *None of the time* | 0-12.5 | Sliding scale | Prim; Sec; FE | 2019; 2020; 2021 |
| | | | *Rarely* | 12.6-37.5 | | | |
| | | | *Some of the time* | 37.6-62.4 | | | |
| | | | *Often* | 62.5-87.4 | | | |
| | | | *All of the time* | 87.5-100 | | | |
| | | ***I've been feeling interested in other people*** | *None of the time* | 0-12.5 | Sliding scale | Prim; Sec; FE | 2019; 2020; 2021 |
| | | | *Rarely* | 12.6-37.5 | | | |
| | | | *Some of the time* | 37.6-62.4 | | | |
| | | | *Often* | 62.5-87.4 | | | |
| | | | *All of the time* | 87.5-100 | | | |
| | | ***I've had energy to spare*** | *None of the time* | 0-12.5 | Sliding scale | Prim; Sec; FE | 2019; 2020; 2021 |
| | | | *Rarely* | 12.6-37.5 | | | |
| | | | *Some of the time* | 37.6-62.4 | | | |
| | | | *Often* | 62.5-87.4 | | | |
| | | | *All of the time* | 87.5-100 | | | |
| | | ***I've been dealing with problems well*** | *None of the time* | 0-12.5 | Sliding scale | Prim; Sec; FE | 2019; 2020; 2021 |
| | | | *Rarely* | 12.6-37.5 | | | |
| | | | *Some of the time* | 37.6-62.4 | | | |
| | | | *Often* | 62.5-87.4 | | | |
| | | | *All of the time* | 87.5-100 | | | |
| | | ***I've been thinking clearly*** | *None of the time* | 0-12.5 | Sliding scale | Prim; Sec; FE | 2019; 2020; 2021 |
| | | | *Rarely* | 12.6-37.5 | | | |
| | | | *Some of the time* | 37.6-62.4 | | | |



Note: A * in the *Matched* or *Year Included* columns indicates that the *Question* or *Label* column contain differences in the * indicated year or survey version

OxWell 2012 Primary

| | | | | | | |
|---|---|---|---|---|---|---|
| | | *Often* | 62.5-87.4 | | | |
| | | *All of the time* | 87.5-100 | | | |
| | ***I've been feeling good about myself*** | *None of the time* | 0-12.5 | Sliding scale | Prim; Sec; FE | 2019; 2020; 2021 |
| | | *Rarely* | 12.6-37.5 | | | |
| | | *Some of the time* | 37.6-62.4 | | | |
| | | *Often* | 62.5-87.4 | | | |
| | | *All of the time* | 87.5-100 | | | |
| | ***I've been feeling confident*** | *None of the time* | 0-12.5 | Sliding scale | Prim; Sec; FE | 2019; 2020; 2021 |
| | | *Rarely* | 12.6-37.5 | | | |
| | | *Some of the time* | 37.6-62.4 | | | |
| | | *Often* | 62.5-87.4 | | | |
| | | *All of the time* | 87.5-100 | | | |
| | ***I've been able to make my own mind up about things*** | *None of the time* | 0-12.5 | Sliding scale | Prim; Sec; FE | 2019; 2020; 2021 |
| | | *Rarely* | 12.6-37.5 | | | |
| | | *Some of the time* | 37.6-62.4 | | | |
| | | *Often* | 62.5-87.4 | | | |
| | | *All of the time* | 87.5-100 | | | |
| | ***I've been feeling loved*** | *None of the time* | 0-12.5 | Sliding scale | Prim; Sec; FE | 2019; 2020; 2021 |
| | | *Rarely* | 12.6-37.5 | | | |
| | | *Some of the time* | 37.6-62.4 | | | |
| | | *Often* | 62.5-87.4 | | | |
| | | *All of the time* | 87.5-100 | | | |
| | ***I've been interested in new things*** | *None of the time* | 0-12.5 | Sliding scale | Prim; Sec; FE | 2019; 2020; 2021 |
| | | *Rarely* | 12.6-37.5 | | | |
| | | *Some of the time* | 37.6-62.4 | | | |
| | | *Often* | 62.5-87.4 | | | |
| | | *All of the time* | 87.5-100 | | | |
| | ***I've been feeling cheerful*** | *None of the time* | 0-12.5 | Sliding scale | Prim; Sec; FE | 2019; 2020; 2021 |
| | | *Rarely* | 12.6-37.5 | | | |
| | | *Some of the time* | 37.6-62.4 | | | |
| | | *Often* | 62.5-87.4 | | | |
| | | *All of the time* | 87.5-100 | | | |
| | ***I've been feeling close to other people*** | *None of the time* | 0-12.5 | Sliding scale | Prim; Sec; FE | 2019; 2020; 2021 |
| | | *Rarely* | 12.6-37.5 | | | |
| | | *Some of the time* | 37.6-62.4 | | | |



Note: A * in the *Matched* or *Year Included* columns indicates that the *Question* or *Label* column contain differences in the * indicated year or survey version

OxWell 2012 Primary

| | | | | | | |
|---|---|---|---|---|---|---|
| | | *Often* | 62.5-87.4 | | | |
| | | *All of the time* | 87.5-100 | | | |
| | ***Overall, are you basically satisfied with your life?*** | *Unsatisfied most of the time* | 0-12.5 | Sliding scale | Prim; Sec; FE | 2019; 2020; 2021 |
| | | *Quite unsatisfied* | 12.6-37.5 | | | |
| | | *Neither satisfied nor unsatisfied* | 37.6-62.4 | | | |
| | | *Quite satisfied* | 62.5-87.4 | | | |
| | | *Satisfied* | 87.5-100 | | | |
| | ***How often have you been so worried about something you can not sleep at night?*** | *Never* | 0-12.5 | Sliding scale | Prim; Sec; FE | 2019; 2020; 2021 |
| | | *Once or twice* | 12.6-37.5 | | | |
| | | *Sometimes* | 37.6-62.4 | | | |
| | | *Most nights* | 62.5-87.4 | | | |
| | | *Every night* | 87.5-100 | | | |
| | ***Thinking about how happy you have felt over the past week, would you say you felt generally*** | *Unhappy most of the time* | 0-12.5 | Sliding scale | Prim; Sec*; FE* | 2019; 2020; 2021* |
| | | *Quite unhappy* | 12.6-37.5 | | | |
| | | *Neither happy nor unhappy* | 37.6-62.4 | | | |
| | | *Quite happy* | 62.5-87.4 | | | |
| | | *Happy most of the time* | 87.5-100 | | | |
| | ***Thinking about how happy you have felt over the past week AT SCHOOL, would you say you felt generally*** | *Unhappy most of the time* | 0-12.5 | Sliding scale | Prim; Sec*; FE* | 2019; 2021* |
| | | *Quite unhappy* | 12.6-37.5 | | | |
| | | *Neither happy nor unhappy* | 37.6-62.4 | | | |
| | | *Quite happy* | 62.5-87.4 | | | |
| | | *Happy most of the time* | 87.5-100 | | | |
| | ***Overall, how confident about your future do you generally feel?*** | Not at all confident | 0-12.5 | Sliding scale | Prim ; Sec; FE | 2019;2021 |
| | | Not very confident | 12.6-37.5 | | | |
| | | Quite confident | 37.6-62.4 | | | |
| | | Confident | 62.5-87.4 | | | |
| | | Extremely confident | 87.5-100 | | | |
| | ***How proud do you feel of things you have achieved in your life?*** | Not at all | 0-12.5 | Sliding scale | Prim ; Sec; FE | 2019; 2021 |
| | | Not very | 12.6-37.5 | | | |
| | | A bit | 37.6-62.4 | | | |
| | | Quite | 62.5-87.4 | | | |



Note: A * in the *Matched* or *Year Included* columns indicates that the *Question* or *Label* column contain differences in the * indicated year or survey version

## OxWell 2012 Primary

| | | | |
|---|---|---|---|
| | | Extremely | 87.5-100 |



Note: A * in the *Matched* or *Year Included* columns indicates that the *Question* or *Label* column contain differences in the * indicated year or survey version

OxWell 2012 Primary

## MENTAL HEALTH SERVICES

| CODE | Contingent | Question | Label | Value | Type | Matched | Year Included |
|---|---|---|---|---|---|---|---|
| | | ***Do you know who helps children in your school when they feel upset and need help (mental health support)?*** | *Yes* | Y | String | Prim*; Sec; FE | 2019*; 2020; 2021 |
| | | | *No* | N | | | |
| | | | *Not sure* | D | | | |
| | | ***Is it easy for you to find someone when you feel upset and need help?*** | *Very difficult* | 0-12.5 | Sliding scale | Prim*; Sec; FE | 2020; 2021* |
| | | | *Quite difficult* | 12.6-37.5 | | | |
| | | | *Sometimes difficult* | 37.6-62.4 | | | |
| | | | *Quite easy* | 62.5-87.4 | | | |
| | | | *Very easy* | 87.5-100 | | | |
| | | ***Who provides mental health support in your school?*** | | | Tick box | Prim*; Sec; FE | 2021 |
| | | | *Class teacher* | Y | | | |
| | | | *Teaching assistants* | Y | | | |
| | | | *Another adult in school* | Y | | | |
| | | | *A mental health worker (from outside school) who visits the school* | Y | | | |
| | | ***If you felt you needed to speak to someone to get mental health support what would you do?*** | | | Tick box | Prim; Sec; FE | 2020*; 2021* |
| | | | *Speak to a parent/carer* | Y | | | |
| | | | *Speak to another family member* | Y | | | |
| | | | *Speak to a friend* | Y | | | |
| | | | *Speak to someone who works at school (e.g. teacher, class assistant or other adults in school)* | Y | | | |
| | | | *Look for help online (e.g. Childline, Mind)* | Y | | | |
| | | | *Speak to another adult I trust (e.g. a mental health worker who visits the school)* | Y | | | |
| | | | *Nothing* | Y | | | |
| | | | *Other* | Y | | | |



Note: A * in the *Matched* or *Year Included* columns indicates that the *Question* or *Label* column contain differences in the * indicated year or survey version

OxWell 2012 Primary

## RELATIONSHIPS

| CODE | Contingent | Question | Label | Value | Type | Matched | Year Included |
|---|---|---|---|---|---|---|---|
| | | ***How easy do you find it to make and keep friends?*** | *Very difficult*<br>*Quite difficult*<br>*Sometimes difficult*<br>*Quite easy*<br>*Very easy* | 0-12.5<br>12.6-37.5<br>37.6-62.4<br>62.5-87.4<br>87.5-100 | String | Prim; Sec; FE | 2019; 2021 |
| | | ***How well do you get along with other people in your household?*** | *Very well or well*<br>*Most of the time well*<br>*Not well or not at all well* | WELL<br>WELLMOST<br>NOTWELL | String | Prim; Sec; FE | 2020; 2021 |
| | | ***How well do you get along with your friends?*** | *Very well or well*<br>*Most of the time well*<br>*Not well or not at all well* | WELL<br>WELLMOST<br>NOTWELL | String | Prim; Sec; FE | 2020; 2021 |
| | | ***How often do you feel that you have no one to talk to (in person/online/phone)?*** | *Hardly ever or never*<br>*Some of the time*<br>*Often* | NEVER<br>SOMETIMES<br>OFTEN | String | Prim; Sec; FE | 2020; 2021 |
| | | ***How often do you feel left out?*** | *Hardly ever or never*<br>*Some of the time*<br>*Often* | NEVER<br>SOMETIMES<br>OFTEN | String | Prim; Sec; FE | 2020; 2021 |
| | | ***How often do you feel alone?*** | *Hardly ever or never*<br>*Some of the time*<br>*Often* | NEVER<br>SOMETIMES<br>OFTEN | String | Prim; Sec; FE | 2021 |
| | | ***How often do you feel lonely?*** | *Hardly ever or never*<br>*Some of the time*<br>*Often* | NEVER<br>SOMETIMES<br>OFTEN | String | Prim; Sec; FE | 2020; 2021 |



Note: A * in the *Matched* or *Year Included* columns indicates that the *Question* or *Label* column contain differences in the * indicated year or survey version

OxWell 2012 Primary

## RESEARCH

| CODE | Contingent | Question | Label | Value | Type | Matched | Year Included |
|---|---|---|---|---|---|---|---|
| | | ***If this survey asked you to tell us your name, so that we would know who you are, would you still take part>?*** | *Yes* | Y | String | Prim; Sec; FE | 2021 |
| | | | *No* | N | | | |
| | | | *Maybe* | MAYBE | | | |
| | | | *I don't understand* | D | | | |
| | | ***Did you also complete this survey last year at school?*** | *Yes* | Y | String | Prim; Sec; FE | 2020; 2021* |
| | | | *No* | N | | | |
| | | | *I can't remember* | NOMEMORY | | | |
| | | ***If this survey asked you to tell us your name, so that we would know who you are, would you still take part?*** | *Not at all* | 0-12.5 | Sliding scale | Prim*; Sec; FE | 2019*; 2020; 2021* |
| | | | *Not much* | 12.6-37.5 | | | |
| | | | *Some* | 37.6-62.4 | | | |
| | | | *Quite a lot* | 62.5-87.4 | | | |
| | | | *A great deal* | 87.5-100 | | | |
| | | ***If this survey asked you to tell us your name, so that we would know who you are, would you have answered any of questions in this survey differently?*** | *Not at all* | 0-12.5 | Sliding scale | Prim*; Sec; FE | 2019*; 2020; 2021* |
| | | | *Not much* | 12.6-37.5 | | | |
| | | | *Some* | 37.6-62.4 | | | |
| | | | *Quite a lot* | 62.5-87.4 | | | |
| | | | *A great deal* | 87.5-100 | | | |



Note: A * in the *Matched* or *Year Included* columns indicates that the *Question* or *Label* column contain differences in the * indicated year or survey version
