# Supplementary material for: Study protocol: the OxWell school survey investigating social, emotional and behavioural factors associated with mental health and well-being
Source: BMJ Open. 2021 Nov 30;11(12):e052717. doi: 10.1136/bmjopen-2021-052717 (PMC9066348; doi:10.1136/bmjopen-2021-052717)
Supplement: Supplementary data [file bmjopen-2021-052717supp004.pdf]

OxWell VARIABLE GUIDE 2021

Questionnaire: FE (Years 12-13)

For the most up-to-date versions, please see https://osf.io/sekhr/

Contents

Variable Guide Table of Contents

DEMOGRAPHICS 3
DIET AND FOOD POVERTY 4
SLEEP 5
EXERCISE 8
SUBSTANCE USE 11
GAMBLING 15
DOMESTIC ABUSE 16
SCHOOL EXPERIENCE 18
SAFETY 24
INTERNET 26
GAMING 28
COVID 29
BULLYING 31
SELF-HARM 32
MENTAL HEALTH 42
ADVERSE CHILDHOOD EXPERIENCES 48
MENTAL HEALTH SERVICES 49
RELATIONSHIPS 57
SEXUAL HEALTH 58
RESEARCH 60
PARANOIA 61

OxWell 2021 FE

Note: A * in the *Matched* or *Year Included* columns indicates that the *Question* or *Label* column contain differences in the * indicated year or survey version

OxWell 2021 FE

DEMOGRAPHICS

CODE	Contingent	Question	Label	Value	Type	Matched	Year Included
		Please enter your Year Group	*Year group*	8 - 11	Numeric	Prim; Sec; FE	2019, 2020, 2021
		Are you male or female?	*Male*<br>*Female*<br>*Other/ Prefer not to answer*	M<br>F<br>R	String	Prim; Sec*; FE*	2019, 2020, 2021*
		How old are you	*Age in full years*	15 – 25+	Numeric	Prim; Sec; FE	2019, 2020, 2021
		Who do you live with?	*I live with both of my parents*<br>*I live with one of my parents*<br>*My parents are separated and I live in two homes*<br>*I live with other relatives or adoptive parents*<br>*I live with other people (foster carers, foster home, childrens home)*<br>*I live in more than one place*<br>*I live somewhere else*<br>*I would rather not say*	PARENTS<br>PARENT<br>SEPARATED PARENT<br>RELATIVE<br>OTHERHOME<br>MANY<br>OTHER<br>RF	String	Prim; Sec; FE	2019*; 2020;2021
		Is your home rented?	*Yes*<br>*No*<br>*Don't know*	Y<br>N<br>R	String	Prim; Sec; FE	2021
		Were you born in the UK	*Yes*<br>*No*<br>*Would rather not say*	Y<br>N<br>R	String	Prim; Sec; FE	2019*;2020; 2021
		Were both of your parents born in the UK?	*Yes*<br>*No*<br>*Would rather not say*	Y<br>N<br>R	String	Prim; Sec; FE	2019*;2020; 2021

Note: A * in the *Matched* or *Year Included* columns indicates that the *Question* or *Label* column contain differences in the * indicated year or survey version

OxWell 2021 FE

DIET AND FOOD POVERTY

CODE	Contingent	Question	Label	Value	Type	Matched	Year Included
		Do you usually eat breakfast?	*Never*<br>*Not often*<br>*Sometimes*<br>*Usually*<br>*Every morning*	0-12.5<br>12.6-37.5<br>37.6-62.4<br>62.5-87.4<br>87.5-100	Sliding scale	Prim; Sec; FE	2019; 2020; 2021
		Some young people go to school or to bed hungry because there is not enough food at home. How often does this happen to you?	*Not at all*<br>*Once or twice*<br>*Sometimes*<br>*Most days*<br>*Every day*	0-12.5<br>12.6-37.5<br>37.6-62.4<br>62.5-87.4<br>87.5-100	Sliding scale	Prim; Sec; FE	2019; 2020; 2021

Note: A * in the *Matched* or *Year Included* columns indicates that the *Question* or *Label* column contain differences in the * indicated year or survey version

OxWell 2021 FE

SLEEP

CODE	Contingent	Question	Label	Value	Type	Matched	Year Included
		How long do you usually take to fall asleep?	*0 mins*<br>*30 mins (half an hour)*<br>*60 mins (1 hour)*<br>*90 mins (1.5 hours)*<br>*120+ mins (2 hours or more)*	0-12.5<br>12.6-37.5<br>37.6-62.4<br>62.5-87.4<br>87.5-100	Sliding scale	Prim; Sec; FE	2021
		What time do you usually go to bed on a school/college night?	*6pm*<br>*8pm*<br>*10pm*<br>*12 midnight*<br>*2am or later*	0-12.5<br>12.6-37.5<br>37.6-62.4<br>62.5-87.4<br>87.5-100	Sliding scale	Prim; Sec; FE	2021
		What time do you usually try to fall asleep on a school/college night?	*6pm*<br>*8pm*<br>*10pm*<br>*12 midnight*<br>*2am or later*	0-12.5<br>12.6-37.5<br>37.6-62.4<br>62.5-87.4<br>87.5-100	Sliding scale	Prim; Sec; FE	2021
		What time do you usually wake up on a school/college day?	*5am*<br>*7am*<br>*9am*<br>*11am*<br>*1pm or later*	0-12.5<br>12.6-37.5<br>37.6-62.4<br>62.5-87.4<br>87.5-100	Sliding scale	Prim; Sec; FE	2021
		What time do you usually go to bed at the weekend?	*6pm*<br>*8pm*<br>*10pm*<br>*12 midnight*<br>*2am or later*	0-12.5<br>12.6-37.5<br>37.6-62.4<br>62.5-87.4<br>87.5-100	Sliding scale	Prim; Sec; FE	2021
		What time do you usually try to fall asleep at the weekend?	*6pm*<br>*8pm*<br>*10pm*<br>*12 midnight*<br>*2am or later*	0-12.5<br>12.6-37.5<br>37.6-62.4<br>62.5-87.4<br>87.5-100	Sliding scale	Prim; Sec; FE	2021
		What time do you usually wake up at the weekend?	*5am*<br>*7am*	0-12.5<br>12.6-37.5	Sliding scale	Prim; Sec; FE	2021

Note: A * in the *Matched* or *Year Included* columns indicates that the *Question* or *Label* column contain differences in the * indicated year or survey version

OxWell 2021 FE

		9am	37.6-62.4			
		11am	62.5-87.4			
		1pm or later	87.5-100			
	People sometimes feel sleepy during the daytime. During your daytime activities, how much of a problem do you have with sleepiness (feeling sleepy, struggling to stay awake)?	*No problem at all*	0-12.5	Sliding scale	Prim; Sec; FE	2021
		A little problem	12.6-37.5			
		More than a little problem	37.6-62.4			
		A big problem	62.5-87.4			
		A very big problem	87.5-100			
	Thinking about the past month, to what extent has poor sleep troubled you in general?	*Not at all*	NOTATALL	String	Prim; Sec; FE	2021
		A little	LITTLE			
		Somewhat	SOMEWHAT			
		Much	MUCH			
		Very much	VERYMUCH			
	Thinking about a typical night in the last month, how many nights a week do you have a problem with your sleep?	*0-1*	0TO1	String	Prim; Sec; FE	2021
		2	2			
		3	3			
		4	4			
		5-7	5TO7			
	On a school/college night (when you have lessons the next day), who usually sets your bedtime?	*Yourself*	SELF	String	Prim; Sec; FE	2021
		Parent/carer/guardian/	CARER			
		other family member	##			
		other	OTHER			
	On a weekend night (no lessons the next day), who usually sets your bedtime?	*Yourself*	SELF	String	Prim; Sec; FE	2021
		Parent/carer/guardian/	CARER			
		other family member	##			
		other	OTHER			
	On school/college nights (when you have lessons the next day), do you have a rule or set time in your house about when you are supposed to turn off or put away computers, phones or other electronics?	*Yes*	Y	String	Prim; Sec; FE	2021
		No	N			
	How often do you use social media (e.g. tik-tok, instagram) in the hour before you intend to go to sleep?	*Never*	0-12.5	Sliding scale	Prim; Sec; FE	2021
		Rarely (1-2 times a month)	12.6-37.5			
		Sometimes (1-2 times a week)	37.6-62.4			
		Often (3-4 times a week)	62.5-87.4			

Note: A * in the *Matched* or *Year Included* columns indicates that the *Question* or *Label* column contain differences in the * indicated year or survey version

OxWell 2021 FE

		Daily	87.5-100			
	How often do you play video games in the hour before you intend to go to sleep (including games on consoles, computer, tablet, mobile phone or other portable gaming device)?	*Never*	0-12.5	Sliding scale	Prim; Sec; FE	2021
		Rarely (1-2 times a month)	12.6-37.5			
		Sometimes (1-2 times a week)	37.6-62.4			
		Often (3-4 times a week)	62.5-87.4			
		Daily	87.5-100			

Note: A * in the *Matched* or *Year Included* columns indicates that the *Question* or *Label* column contain differences in the * indicated year or survey version

OxWell 2021 FE

EXERCISE

CODE	Contingent	Question	Label	Value	Type	Matched	Year Included
		About how many hours physical activity or exercise do you do over a whole week (in and out of school/college, during term-time)	*1 hour or less)*<br>*3 hours*<br>*6 hours*<br>*9 hours*<br>*12 hours or more*	0-12.5<br>12.6-37.5<br>37.6-62.4<br>62.5-87.4<br>87.5-100	Sliding scale	Prim; Sec; FE	2020; 2021*
		How many times per week do you normally do PE/sports during school/college time?	*0*<br>*3*<br>*6*<br>*9*<br>*12 or more*	0-12.5<br>12.6-37.5<br>37.6-62.4<br>62.5-87.4<br>87.5-100	Sliding scale	Prim; Sec; FE	2021
		How many times per week do you do sports or exercise outside of school/college hours?	*0*<br>*3*<br>*6*<br>*9*<br>*12 or more*	0-12.5<br>12.6-37.5<br>37.6-62.4<br>62.5-87.4<br>87.5-100	Sliding scale	Prim; Sec; FE	2021
		Is most of your exercise at school/college or outside of school/college?	*Only at*<br>*Mostly at*<br>*Same in/out of*<br>*Mostly outside*<br>*All outside*	0-12.5<br>12.6-37.5<br>37.6-62.4<br>62.5-87.4<br>87.5-100	Sliding scale	Prim; Sec; FE	2021
		Compared to before the first lockdown, how much time do you spend doing sports or exercise now?	*Much less*<br>*Slightly less*<br>*The same amount*<br>*Slightly more*<br>*Much more*	0-12.5<br>12.6-37.5<br>37.6-62.4<br>62.5-87.4<br>87.5-100	Sliding scale	Prim; Sec; FE	2021
		How much do you enjoy physical exercise outside of school/college time?	*Never*<br>*Not often*<br>*Sometimes*<br>*Quite often*<br>*Always*	0-12.5<br>12.6-37.5<br>37.6-62.4<br>62.5-87.4<br>87.5-100	Sliding scale	Prim; Sec; FE	2021
		Would you like to do more sports/exercise?	*Yes*<br>*No*	Y<br>N	String	Prim; Sec; FE	2020; 2021

Note: A * in the *Matched* or *Year Included* columns indicates that the *Question* or *Label* column contain differences in the * indicated year or survey version

OxWell 2021 FE

	How much do you normally enjoy your PE lessons?	*Never*	0-12.5	String	Prim; Sec; FE	2020; 2021
		Not often	12.6-37.5			
		Sometimes	37.6-62.4			
		Quite often	62.5-87.4			
		Always	87.5-100			
	How important are the following in your decision to do sport or physical activity:					
	Spend time with friends and meet new people	*Not at all important*	0-12.5	Sliding scale	Prim; Sec; FE	2019; 2021
		Not very important	12.6-37.5			
		Quite important	37.6-62.4			
		Important	62.5-87.4			
		Very important	87.5-100			
	Exercise get fit	*Not at all important*	0-12.5	Sliding scale	Prim; Sec; FE	2019; 2020; 2021
		Not very important	12.6-37.5			
		Quite important	37.6-62.4			
		Important	62.5-87.4			
		Very important	87.5-100			
	Unwind/reduce stress	*Not at all important*	0-12.5	Sliding scale	Prim; Sec; FE	2019; 2020; 2021
		Not very important	12.6-37.5			
		Quite important	37.6-62.4			
		Important	62.5-87.4			
		Very important	87.5-100			
	Weight management/body shaping	*Not at all important*	0-12.5	Sliding scale	Prim; Sec; FE	2019; 2020; 2021
		Not very important	12.6-37.5			
		Quite important	37.6-62.4			
		Important	62.5-87.4			
		Very important	87.5-100			
	Learn a new skill	*Not at all important*	0-12.5	Sliding scale	Prim; Sec; FE	2019; 2020; 2021
		Not very important	12.6-37.5			
		Quite important	37.6-62.4			
		Important	62.5-87.4			
		Very important	87.5-100			
	Improve performance/do well competitively	*Not at all important*	0-12.5	Sliding scale	Prim; Sec; FE	2019; 2020; 2021
		Not very important	12.6-37.5			
		Quite important	37.6-62.4			
		Important	62.5-87.4			
		Very important	87.5-100			
	Enjoyment - feeling good physically/mentally	*Not at all important*	0-12.5	Sliding scale	Prim; Sec; FE	2019; 2020; 2021
		Not very important	12.6-37.5			
		Quite important	37.6-62.4			
		Important	62.5-87.4			

Note: A * in the *Matched* or *Year Included* columns indicates that the *Question* or *Label* column contain differences in the * indicated year or survey version

OxWell 2021 FE

		Very important	87.5-100			
	Have to do it for others - school/college/team/family	*Not at all important*	0-12.5	Sliding scale	Prim; Sec; FE	2019; 2020; 2021
		Not very important	12.6-37.5			
		Quite important	37.6-62.4			
		Important	62.5-87.4			
		Very important	87.5-100			

Note: A * in the *Matched* or *Year Included* columns indicates that the *Question* or *Label* column contain differences in the * indicated year or survey version

OxWell 2021 FE

SUBSTANCE USE

CODE	Contingent	Question	Label	Value	Type	Matched	Year Included
		Do you smoke cigarettes (i.e. tobacco, NOT vaping/e-cigarettes)?	*Never*	0-12.5	Sliding scale	Sec*; FE	2019; 2020*
			Not often (once or twice)	12.6-37.5			
			Sometimes (monthly)	37.6-62.4			
			Quite often (weekly)	62.5-87.4			
			Most days	87.5-100			
		Do you use e-cigarettes (vaping)?	*Never*	0-12.5	Sliding scale	Sec*; FE	2019; 2020*
			Not often (once or twice)	12.6-37.5			
			Sometimes (monthly)	37.6-62.4			
			Quite often (weekly)	62.5-87.4			
			Most days	87.5-100			
		Have you ever had an alcoholic drink?	*Never*	0-12.5	Sliding scale	Sec*; FE	2019; 2020*
			Not often (once or twice)	12.6-37.5			
			Sometimes (monthly)	37.6-62.4			
			Quite often (weekly)	62.5-87.4			
			Most days	87.5-100			
		When did you last have an alcoholic drink?	*Today*	TODAY	String	Sec; FE	2020
			Yesterday	YESTERDAY			
			Some other time in the last 7 days	LAST7DAYS			
			1 week, but less than 2 weeks ago	1TO2WEEKS			
			2 weeks, but less than 4 weeks ago	2TO4WEEKS			
			1 month, but less than 6 months ago	1TO6MONTHS			
			6 months ago or more	GT6MONTHS			
		Have you ever been drunk?	*Never*	0-12.5	Sliding scale	Sec*; FE	2019; 2020*
			Once	12.6-37.5			
			2-3 times	37.6-62.4			
			4-10 times	62.5-87.4			
			More than 10 times	87.5-100			
		Have you been drunk in the last 7 days?	*Yes*	Y	String	Sec; Fe	2020
			No	N			

Note: A * in the *Matched* or *Year Included* columns indicates that the *Question* or *Label* column contain differences in the * indicated year or survey version

OxWell 2021 FE

	When you drink alcohol, where are you USUALLY?	*At home*<br>*At someone else's home*<br>*At a party*<br>*In a pub or a bar In a club, disco or gig/festival*<br>*At school*<br>*Out on the street, in a park or other outdoor area*<br>*Somewhere else*	HOME<br>HOMEELSE<br>PARTY<br>PUB<br>SCHOOL<br>OUTDOOR<br>ELSEWHERE	String	Sec; Fe	2020
	Have you ever been offered something to get you high or drugs not prescribed by your doctor (examples are illegal drugs, legal highs and prescription medication)?	*Yes*<br>*No*	Y<br>N	String	Sec*; FE	2019; 2020*
	Have you ever taken something to get high or self-medicated with drugs not prescribed by your doctor (examples are illegal drugs, legal highs and prescription medication)?	*Never*<br>*Yes – Once*<br>*Yes – On more than one occasion*	N<br>YONCE<br>YMORE	String	Sec*; FE	2019*; 2020*
	The first time you used drugs, how did you get them?	*From someone I know from school*<br>*From someone I know who is not from my school*<br>*From a friend of a friend*<br>*From a stranger*<br>*From a website/online/internet*	SCHOOLFRIEND<br>NOTSCHOOLFRIEND<br>FRIENDSFRIEDS<br>STRANGER<br>ONLINE	String	Sec; FE	2020
	Where were you the first time you used drugs?	*At home*<br>*At someone else's home*<br>*At a party*<br>*In a pub or a bar In a club, disco or gig/festival*<br>*At school*<br>*Out on the street, in a park or other outdoor area*<br>*Somewhere else*	HOME<br>HOMEELSE<br>PARTY<br>PUB<br>SCHOOL<br>OUTDOOR<br>ELSEWHERE	String	Sec; FE	2021
	Where were you the last time you used drugs?	*At home*<br>*At someone else's home*	HOME<br>HOMEELSE	String	Sec; FE	2021

Note: A * in the *Matched* or *Year Included* columns indicates that the *Question* or *Label* column contain differences in the * indicated year or survey version

OxWell 2021 FE

		At a party	PARTY			
		In a pub or a bar In a club, disco or gig/festival	PUB			
		At school	SCHOOL			
		Out on the street, in a park or other outdoor area	OUTDOOR			
		Somewhere else	ELSEWHERE			
	The last time you used drugs, did you use them all yourself or did you sell or give some to someone else?	*I used it all myself*	SELF	String	Sec; FE	2021
		I sold some of it	SOLDSOME			
		I gave some of it away	GIFTED			
		I both gave some away and sold some	SOLDANDGIFTED			
	When was the last time you ever used or took any of the following?			String	Sec; FE	2021
	Cannabis (weed, resin, skunk, CBD)	*In the last months*	THISMONTH			
		In the last year	THISYEAR			
		More than a year ago	GTYEAR			
		Never	N			
	Nitrous Oxide (NO, NOS, N2O, laughing gas, balloons)	*In the last months*	THISMONTH			
		In the last year	THISYEAR			
		More than a year ago	GTYEAR			
		Never	N			
	Ketamine (ket, special K, keta, gummies)	*In the last months*	THISMONTH			
		In the last year	THISYEAR			
		More than a year ago	GTYEAR			
		Never	N			
	Ecstasy/MDMA (E, eccies, mandy, molly)	*In the last months*	THISMONTH			
		In the last year	THISYEAR			
		More than a year ago	GTYEAR			
		Never	N			
	Benzodiazepines (benzos, vallies, diazepam, xanax,etizolam)	*In the last months*	THISMONTH			
		In the last year	THISYEAR			
		More than a year ago	GTYEAR			
		Never	N			
	Other (amphetamines like speed, cocaine, mephedrone, legal highs, psychedelics like LSD, acid, magic mushrooms, psilocybin, DMT, 2C, opioids like Fentanyl or Heroin)	*In the last months*	THISMONTH			
		In the last year	THISYEAR			
		More than a year ago	GTYEAR			
		Never	N			

Note: A * in the *Matched* or *Year Included* columns indicates that the *Question* or *Label* column contain differences in the * indicated year or survey version

OxWell 2021 FE

	How easy would it be for you to get illegal drugs if you wanted to?	*Very difficult*	0-12.5	Sliding scale	Sec; FE	2020; 2021
		Difficult	12.6-37.5			
		Neither difficult nor easy	37.6-62.4			
		Quite easy	62.5-87.4			
		Very easy	87.5-100			
	Have you ever been encouraged or felt pressured to sell drugs?	*No - I have not been encouraged or pressured*	N	String	Sec; FE	2021
		Yes - I have been pressured	YPRESSURED			
		Yes - I have been encouraged	YENCOURAGED			
	If you wanted information about drugs, who/where would you go to?			Tick box	Sec; FE	2021
		Teacher	Y			
		Parent(s)/Carer	Y			
		Friend(s)	Y			
		Drug service	Y			
		Search engine (e.g. Google)	Y			
		A specific internet site (Talk to Frank, Know the Score)	Y			
		An internet site, but not one of the ones listed above	Y			
		Don't know	Y			

Note: A * in the *Matched* or *Year Included* columns indicates that the *Question* or *Label* column contain differences in the * indicated year or survey version

OxWell 2021 FE

GAMBLING

CODE	Contingent	Question	Label	Value	Type	Matched	Year Included
		Have you ever tried online gambling?	*Yes*	Y	String	Sec*; FE	2020; 2021
			No	N			
		Have you ever used real money for online gambling?	*Never*	0-12.5	Sliding scale	Sec*; FE	2020; 2021
			Once or twice	12.6-37.5			
			A few times	37.6-62.4			
			Weekly	62.5-87.4			
			Daily	87.5-100			
		Do you owe money to someone who isn't family?	*No*	0-12.5	Sliding scale	Sec*; FE	2020; 2021
			Yes – a little	12.6-37.5			
			Yes – a lot	37.6-62.4			

Note: A * in the *Matched* or *Year Included* columns indicates that the *Question* or *Label* column contain differences in the * indicated year or survey version

OxWell 2021 FE

DOMESTIC ABUSE

CODE	Contingent	Question	Label	Value	Type	Matched	Year Included
		Physical Abuse: Did a parent or other adult in the household hit, beat, kick or physically try to hurt you in any way?		Y	Tick box	Sec; FE	2021
		Yes, it has happened in my life	*Once or twice*	YONCE	String	Sec; FE	2021
			Many times	YMANY			
		Yes, it has happened in the past twelve months	*Once or twice*	YONCE	String	Sec; FE	2021
			Many times	YMANY			
		Emotional abuse: Did a parent or other adult in the household swear at you, insult you, humiliate you, threaten you or make you feel unwanted?		Y	Tick box	Sec; FE	2021
		Yes, it has happened in my life	*Once or twice*	YONCE	String	Sec; FE	2021
			Many times	YMANY			
		Yes, it has happened in the past twelve months	*Once or twice*	YONCE	String	Sec; FE	2021
			Many times	YMANY			
		Sexual abuse: Did someone at least five years older than you or an adult touch or fondle you or have you touch their body in a sexual way?		Y	Tick box	Sec; FE	2021
		Yes, it has happened in my life	*Once or twice*	YONCE	String	Sec; FE	2021
			Many times	YMANY			
		Yes, it has happened in the past twelve months	*Once or twice*	YONCE	String	Sec; FE	2021
			Many times	YMANY			
		Physical neglect: Did your parent/caregiver for long periods of time not provide you with enough food or drink, clean clothes, or a clean and warm place to live?		Y	Tick box	Sec; FE	2021
		Yes, it has happened in my life	*Once or twice*	YONCE	String	Sec; FE	2021
			Many times	YMANY			
		Yes, it has happened in the past twelve months	*Once or twice*	YONCE	String	Sec; FE	2021
			Many times	YMANY			
		Emotional neglect: Were there times when there was no adult living with you who made you feel loved?		Y	Tick box	Sec; FE	2021
		Yes, it has happened in my life	*Once or twice*	YONCE	String	Sec; FE	2021
			Many times	YMANY			
		Yes, it has happened in the past twelve months	*Once or twice*	YONCE	String	Sec; FE	2021
			Many times	YMANY			
		Witnessing parental violence: Did you see or hear one of your parents/carers being slapped, kicked, punched, beaten or deliberately hurt by a partner or ex		Y	Tick box	Sec; FE	2021
		Yes, it has happened in my life	*Once or twice*	YONCE	String	Sec; FE	2021
			Many times	YMANY			
		Yes, it has happened in the past twelve months	*Once or twice*	YONCE	String	Sec; FE	2021

Note: A * in the *Matched* or *Year Included* columns indicates that the *Question* or *Label* column contain differences in the * indicated year or survey version

OxWell 2021 FE

		Many times	YMANY			
	Have you ever been a victim of abuse from a friend/peer/boyfriend/ girlfriend?	*Never*	0-12.5	Sliding scale	Sec; FE	2019; 2021
		Not often	12.6-37.5			
		Sometimes	37.6-62.4			
		Quite often	62.5-87.4			
		Most days	87.5-100			
	Is this abuse still happening?	*Yes*	Y	String	Sec; FE	2019; 2021
		No	N			
	How old is he/she?	*About the same age*	SAME	String	Sec; FE	2019; 2021
		Younger	YOUNGER			
		Older	OLDER			
		An adult	ADULT			
		Would rather not say	R			

Note: A * in the *Matched* or *Year Included* columns indicates that the *Question* or *Label* column contain differences in the * indicated year or survey version

OxWell 2021 FE

SCHOOL EXPERIENCE

CODE	Contingent	Question	Label	Value	Type	Matched	Year Included
		How much help and support with learning do you feel that you get at school/college?	*No help at all*	0-12.5	Sliding scale	Prim; Sec; FE	2019; 2020; 2021
			Not enough help	12.6-37.5			
			Just about enough help	37.6-62.4			
			Most of the help I need	62.5-87.4			
			All of the help I need	87.5-100			
		How much help and support do you feel you get with your homework from the people you live with?	*No help at all*	0-12.5	Sliding scale	Prim; Sec; FE	2019; 2020; 2021
			Not enough help	12.6-37.5			
			Just about enough help	37.6-62.4			
			Most of the help I need	62.5-87.4			
			All of the help I need	87.5-100			
		How would you describe being at your school/college?					2019*; 2021
		I enjoy my school/college	*Strongly Disagree*	0-12.5	Sliding scale	Prim; Sec; FE	2019; 2021
			Disagree	12.6-37.5			
			Neither Agree nor disagree	37.6-62.4			
			Agree	62.5-87.4			
			Strongly Agree	87.5-100			
		I try my best at school/college	*Strongly Disagree*	0-12.5	Sliding scale	Prim; Sec; FE	2019; 2021
			Disagree	12.6-37.5			
			Neither Agree nor disagree	37.6-62.4			
			Agree	62.5-87.4			
			Strongly Agree	87.5-100			
		I learn a lot at school/college	*Strongly Disagree*	0-12.5	Sliding scale	Prim; Sec; FE	2019; 2021
			Disagree	12.6-37.5			
			Neither Agree nor disagree	37.6-62.4			
			Agree	62.5-87.4			
			Strongly Agree	87.5-100			
		My school/college is giving me useful skills and knowledge	*Strongly Disagree*	0-12.5	Sliding scale	Prim; Sec; FE	2019; 2021
			Disagree	12.6-37.5			
			Neither Agree nor disagree	37.6-62.4			
			Agree	62.5-87.4			
			Strongly Agree	87.5-100			
		My school/college has lots of activities (like sport and drama) to take part in at lunchtime or after school/college	*Strongly Disagree*	0-12.5	Sliding scale	Prim; Sec; FE	2019; 2021
			Disagree	12.6-37.5			
			Neither Agree nor disagree	37.6-62.4			

Note: A * in the *Matched* or *Year Included* columns indicates that the *Question* or *Label* column contain differences in the * indicated year or survey version

OxWell 2021 FE

		Agree	62.5-87.4			
		Strongly Agree	87.5-100			
	My teacher(s) make my lessons fun and interesting	*Strongly Disagree*	0-12.5	Sliding scale	Prim; Sec; FE*	2019; 2021
		Disagree	12.6-37.5			
		Neither Agree nor disagree	37.6-62.4			
		Agree	62.5-87.4			
		Strongly Agree	87.5-100			
	My teacher(s) tell me how I am doing with my work	*Strongly Disagree*	0-12.5	Sliding scale	Prim; Sec; FE*	2019; 2021
		Disagree	12.6-37.5			
		Neither Agree nor disagree	37.6-62.4			
		Agree	62.5-87.4			
		Strongly Agree	87.5-100			
	Other students often disrupt my lessons	*Strongly Disagree*	0-12.5	Sliding scale	Prim; Sec; FE|*	2019; 2021
		Disagree	12.6-37.5			
		Neither Agree nor disagree	37.6-62.4			
		Agree	62.5-87.4			
		Strongly Agree	87.5-100			
	I get enough help at school/college with learning	*Strongly Disagree*	0-12.5	Sliding scale	Prim; Sec; FE	2019; 2021
		Disagree	12.6-37.5			
		Neither Agree nor disagree	37.6-62.4			
		Agree	62.5-87.4			
		Strongly Agree	87.5-100			
	I get enough help at school/college with emotional/pastoral needs	*Strongly Disagree*	0-12.5	Sliding scale	Prim; Sec*; FE*	2019; 2021
		Disagree	12.6-37.5			
		Neither Agree nor disagree	37.6-62.4			
		Agree	62.5-87.4			
		Strongly Agree	87.5-100			
	I feel stressed by school/college work	*Strongly Disagree*	0-12.5	Sliding scale	Prim; Sec; FE	2019; 2021
		Disagree	12.6-37.5			
		Neither Agree nor disagree	37.6-62.4			
		Agree	62.5-87.4			
		Strongly Agree	87.5-100			
	I worry about going to school/college	*Strongly Disagree*	0-12.5	Sliding scale	Prim; Sec; FE	2019; 2021
		Disagree	12.6-37.5			
		Neither Agree nor disagree	37.6-62.4			
		Agree	62.5-87.4			
		Strongly Agree	87.5-100			

Note: A * in the *Matched* or *Year Included* columns indicates that the *Question* or *Label* column contain differences in the * indicated year or survey version

OxWell 2021 FE

	I am often in trouble	*Strongly Disagree*	0-12.5	Sliding scale	Prim; Sec; FE	2019; 2021
		Disagree	12.6-37.5			
		Neither Agree nor disagree	37.6-62.4			
		Agree	62.5-87.4			
		Strongly Agree	87.5-100			
	I am often aggressive or violent	*Strongly Disagree*	0-12.5	Sliding scale	Prim; Sec; FE	2019; 2021
		Disagree	12.6-37.5			
		Neither Agree nor disagree	37.6-62.4			
		Agree	62.5-87.4			
		Strongly Agree	87.5-100			
	I usually achieve top marks at my school/college	*Strongly Disagree*	0-12.5	Sliding scale	Prim; Sec; FE	2019; 2021
		Disagree	12.6-37.5			
		Neither Agree nor disagree	37.6-62.4			
		Agree	62.5-87.4			
		Strongly Agree	87.5-100			
	I am happy to use the school/college toilets	*Strongly Disagree*	0-12.5	Sliding scale	Prim; Sec; FE	2019; 2021
		Disagree	12.6-37.5			
		Neither Agree nor disagree	37.6-62.4			
		Agree	62.5-87.4			
		Strongly Agree	87.5-100			
	About speaking out:			String	Prim; Sec; FE	2021
	Adults in my school/college ask for my opinion on things	*Never/ Rarely*	NEVER			
		Sometimes	SOMETIMES			
		Often	OFTEN			
	Adults in my school/college listen when I share my opinion	*Never/ Rarely*	NEVER			
		Sometimes	SOMETIMES			
		Often	OFTEN			
	My school/college friends ask my advice when they have a problem	*Never/ Rarely*	NEVER			
		Sometimes	SOMETIMES			
		Often	OFTEN			
	If I see something wrong in school/college I feel I can tell someone and they will listen	*Never/ Rarely*	NEVER			
		Sometimes	SOMETIMES			
		Often	OFTEN			
	I can speak up in class when I have a comment or question	*Never/ Rarely*	NEVER			
		Sometimes	SOMETIMES			
		Often	OFTEN			
	I can speak up when I see someone else being hurt	*Never/ Rarely*	NEVER			

Note: A * in the *Matched* or *Year Included* columns indicates that the *Question* or *Label* column contain differences in the * indicated year or survey version

OxWell 2021 FE

		Sometimes	SOMETIMES			
		Often	OFTEN			
	I can ask adults in my school/college for help when I need it	*Never/ Rarely*	NEVER			
		Sometimes	SOMETIMES			
		Often	OFTEN			
	How much do you agree with the following statement: I identify with my school/college community	*Fully disagree*	FULLDISAGREE	String	Prim; Sec; FE	2021
		Disagree	DISAGREE			
		Somewhat disagree	SOMEDISAGREE			
		Neither agree nor disagree	NEITHER			
		Somewhat agree	SOMEAGREE			
		Agree	AGREE			
		Fully agree	FULLAGREE			
	Do you feel your teachers have high expectations of you?	*Never*	NEVER	String	Sec; FE	2021
		Rarely	RARELY			
		Sometimes	SOMETIMES			
		Often	OFTEN			
	Over this school/college year, do you feel you have often been unfairly picked on by a teacher?	*Yes*	Y	String	Sec; FE	2021
		No	N			
	At school/college, are you often told off or punished for things you have not done?	*Never*	NEVER	String	Sec; FE	2021
		Rarely	RARELY			
		Sometimes	SOMETIMES			
		Often	OFTEN			
	Have you received information from school/college that enables you to tell whether a friendship or relationship is abusive?	*Yes*	Y	String	Sec; Fe	2019; 2021
		No	N			
		Don't know	R			
	How many secondary schools have you attended?	*Just one*	1	String	Sec; FE	2021
		2	2			
		3 or more	3ORMORE			
	During this academic year, have you ever been asked to leave your place of learning because of your behaviour?	*No*	N	String	Sec; FE	2021
		Once or twice this year	1TO2			

Note: A * in the *Matched* or *Year Included* columns indicates that the *Question* or *Label* column contain differences in the * indicated year or survey version

OxWell 2021 FE

		Several times this year<br>*Frequently this year*	SEVERAL<br>FREQUENTL Y			
	Where did you go to last time you were asked to leave your place of learning?	Outside the classroom (e.g. corridor)<br>A senior teacher's office<br>A dedicated area that is used for students who have been sent out of the class<br>Another place in school used to isolate/separate students<br>I went home<br>Other	Y<br>Y<br>Y<br>Y<br>Y<br>Y	Tick box	Sec; FE	2021
	What impact did being asked to leave the classroom have on you?	I found it helpful<br>I found it unhelpful<br>No impact	HELPFUL<br>NOTHELPFU L<br>NO	String	Sec; FE	2021
	During this academic year, have you ever been given a detention?	*No*<br>*Once or twice this year*<br>*Several times this year*<br>*Frequently this year*	N<br>1TO2<br>SEVERAL<br>FREQUENTL Y	String	Sec; FE	2021
	Have you ever been excluded from school/college?	*Yes*<br>*No*<br>*Don't know what this means*	Y<br>N<br>D	String	Sec; FE	2021
	Was this ever a permanent exclusion?	*Yes*<br>*No*<br>*Don't know what this means*	Y<br>N<br>D	String	Sec; FE	2021
	In year 11, were you able to get all the information you needed to give a clear picture of the full range of choices available to you after year 11?	*Yes*<br>*No*	Y<br>N	String	FE	2021
	Do you expect to continue in education or training next year?	*Yes*<br>*No*	Y<br>N	String	FE	2021

Note: A * in the *Matched* or *Year Included* columns indicates that the *Question* or *Label* column contain differences in the * indicated year or survey version

OxWell 2021 FE

		Don't know	D			
	**If you need help to plan for your future where would you get this from?**			Tick box	FE	2021
		Websites	Y			
		Information from school/college	Y			
		Careers advisor	Y			
		Careers fair	Y			
		School/College staff	Y			
		Someone outside of school/college	Y			
		Your family/carers/the people you live with	Y			
		Other	Y			
		Not sure where to get advice	Y			

Note: A * in the *Matched* or *Year Included* columns indicates that the *Question* or *Label* column contain differences in the * indicated year or survey version

OxWell 2021 FE

SAFETY

CODE	Contingent	Question	Label	Value	Type	Matched	Year Included
		When you are at school/college, how safe do you feel?	*Very unsafe*	0-12.5	Sliding scale	Prim; Sec; FE	2019; 2020; 2021
			Unsafe	12.6-37.5			
			Neither safe nor unsafe	37.6-62.4			
			Safe	62.5-87.4			
			Very safe	87.5-100			
		How safe do you feel at home or the place where you live?	*Very unsafe*	0-12.5	Sliding scale	Prim; Sec; FE	2019; 2020; 2021
			Unsafe	12.6-37.5			
			Neither safe nor unsafe	37.6-62.4			
			Safe	62.5-87.4			
			Very safe	87.5-100			
		How safe do you from crime?	*Very unsafe*	0-12.5	Sliding scale	Prim	2019; 2021
			Unsafe	12.6-37.5			
			Neither safe nor unsafe	37.6-62.4			
			Safe	62.5-87.4			
			Very safe	87.5-100			
		Some young people feel worried about things happening around them. To what extent do you worry about the following topics:					
		Having enough money to pay for food or living costs	*Not at all worried*	0-12.5	Sliding scale	Sec; FE	2020; 2021
			Not very worried	12.6-37.5			
			Quite worried	37.6-62.4			
			Worried	62.5-87.4			
			Extremely worried	87.5-100			
		Doing well at school/college	*Not at all worried*	0-12.5	Sliding scale	Sec; FE	2020; 2021
			Not very worried	12.6-37.5			
			Quite worried	37.6-62.4			
			Worried	62.5-87.4			
			Extremely worried	87.5-100			
		The climate/environment	*Not at all worried*	0-12.5	Sliding scale	Sec; FE	2020; 2021
			Not very worried	12.6-37.5			
			Quite worried	37.6-62.4			
			Worried	62.5-87.4			
			Extremely worried	87.5-100			
		Not being able to sit key school/college examinations	*Not at all worried*	0-12.5	Sliding scale	Sec; FE	2020; 2021
			Not very worried	12.6-37.5			
			Quite worried	37.6-62.4			

Note: A * in the *Matched* or *Year Included* columns indicates that the *Question* or *Label* column contain differences in the * indicated year or survey version

OxWell 2021 FE

		Worried	62.5-87.4			
		Extremely worried	87.5-100			
	My appearance (how I look and what I wear)	*Not at all worried*	0-12.5	Sliding scale	Sec; FE	2020; 2021
		Not very worried	12.6-37.5			
		Quite worried	37.6-62.4			
		Worried	62.5-87.4			
		Extremely worried	87.5-100			
	How often in the last year have you stolen any money or property that someone was holding, carrying or wearing at the time?	*Not at all*	NO	String	FE	2021
		Once	ONCE			
		2 - 5 times	2TO5			
		More than 6 times	MORETHAN 6			
	How often in the last year have you hit, kicked or punched someone else on purpose with the intention of really hurting them?	*Not at all*	NO	String	FE	2021
		Once	ONCE			
		2 - 5 times	2TO5			
		More than 6 times	MORETHAN 6			
	How often in the last year have you carried a knife or other weapon with you for protection or in case it was needed in a fight?	*Not at all*	NO	String	FE	2021
		Once	ONCE			
		2 - 5 times	2TO5			
		More than 6 times	MORETHAN 6			

Note: A * in the *Matched* or *Year Included* columns indicates that the *Question* or *Label* column contain differences in the * indicated year or survey version

OxWell 2021 FE

INTERNET

CODE	Contingent	Question	Label	Value	Type	Matched	Year Included
		Have you ever posted or done anything on the internet that you later regretted?	*Yes*	Y	String	Sec; FE	2020; 2021
			No	N			
			Would rather not say	R			
		How often has this happened?	*Never*	0-12.5	Sliding scale	Sec; FE	2020; 2021
			Once or twice	12.6-37.5			
			A few times	37.6-62.4			
			Weekly	62.5-87.4			
			Daily	87.5-100			
		Which of the following was this:			Tick box	Sec; FE	2020; 2021
			Posting a photo/video of yourself	Y			
			Posting a photo/video of someone else	Y			
			Writing a public comment	Y			
			Writing a private comment	Y			
			Sending money to someone	Y			
			Buying or doing something that might be illegal	Y			
		About how many hours a day do you usually spend on social media?	*0 hours*	0-12.5	Sliding scale	Sec; FE	2021
			1 hour	12.6-37.5			
			2 hours	37.6-62.4			
			3 hours	62.5-87.4			
			4 hours or more	87.5-100			
		About how many hours a day do you usually play games on an electronic device (e.g. computer, game console or phone)	*0 hours*	0-12.5	Sliding scale	Sec; FE	2021
			1 hour	12.6-37.5			
			2 hours	37.6-62.4			
			3 hours	62.5-87.4			
			4 hours or more	87.5-100			
		About how many hours a day do you usually play games on a computer or games console (PlayStation, Xbox, Nintendo Wii, etc.) in your free time?	*0 hours*	0-12.5	Sliding scale	Sec; FE	2021
			1 hour	12.6-37.5			
			2 hours	37.6-62.4			

Note: A * in the *Matched* or *Year Included* columns indicates that the *Question* or *Label* column contain differences in the * indicated year or survey version

OxWell 2021 FE

		3 hours	62.5-87.4			
		4 hours or more	87.5-100			
	About how many hours a day do you usually play games on your phone in your free time?	*0 hours*	0-12.5	Sliding scale	Sec; FE	2021
		1 hour	12.6-37.5			
		2 hours	37.6-62.4			
		3 hours	62.5-87.4			
		4 hours or more	87.5-100			

Note: A * in the *Matched* or *Year Included* columns indicates that the *Question* or *Label* column contain differences in the * indicated year or survey version

OxWell 2021 FE

GAMING

CODE	Contingent	Question	Label	Value	Type	Matched	Year Included
		About gaming, how often in the last six months:					
		did you think about playing a game all day long?	*Never*	NEVER	String	Sec; FE	2021
			Rarely	RARELY			
			Sometimes	SOMETIMES			
			Often	OFTEN			
			Very often	VERYOFTEN			
		did you spend increasing amounts of time on games?	*Never*	NEVER	String	Sec; FE	2021
			Rarely	RARELY			
			Sometimes	SOMETIMES			
			Often	OFTEN			
			Very often	VERYOFTEN			
		did you play games to forget about real life?	*Never*	NEVER	String	Sec; FE	2021
			Rarely	RARELY			
			Sometimes	SOMETIMES			
			Often	OFTEN			
			Very often	VERYOFTEN			
		have others unsuccessfully tried to reduce your game use?	*Never*	NEVER	String	Sec; FE	2021
			Rarely	RARELY			
			Sometimes	SOMETIMES			
			Often	OFTEN			
			Very often	VERYOFTEN			
		have you felt bad when you were unable to play?	*Never*	NEVER	String	Sec; FE	2021
			Rarely	RARELY			
			Sometimes	SOMETIMES			
			Often	OFTEN			
			Very often	VERYOFTEN			
		did you have fights with others (e.g., family, friends) over your time spent on games?	*Never*	NEVER	String	Sec; FE	2021
			Rarely	RARELY			
			Sometimes	SOMETIMES			
			Often	OFTEN			
			Very often	VERYOFTEN			
		have you neglected other important activities (e.g., school/college, work, sports) to play games?	*Never*	NEVER	String	Sec; FE	2021
			Rarely	RARELY			
			Sometimes	SOMETIMES			
			Often	OFTEN			
			Very often	VERYOFTEN			
		Have you ever spent money on in-game purchases?	*Yes*	Y	String	Sec; FE	2021

Note: A * in the *Matched* or *Year Included* columns indicates that the *Question* or *Label* column contain differences in the * indicated year or survey version

OxWell 2021 FE

CODE	Contingent	Question	Label	Value	Type	Matched	Year Included
			No	N			
		How often do you spend money on in-game purchases?	*Never*<br>*Once*<br>*Couple of times*<br>*Few times a week*<br>*Every day*<br>*All the time*	NEVER<br>ONCE<br>TWICE<br>FEWTIMES<br>WEEK<br>EVERYDAY<br>CONSTANT	String	Sec; FE	2021

COVID

CODE	Contingent	Question	Label	Value	Type	Matched	Year Included
		Do you think you have had Covid?	*No*<br>*Possibly*<br>*Probably*<br>*Yes (confirmed by test)*	N<br>POSSIBLY<br>PROBABLY<br>Y	String	Prim; Sec; FE	2021
		Compared to before the first lockdown, how easy have you found it to remember things?	*Very difficult*<br>*Quite difficult*<br>*The same*<br>*Quite easy*<br>*Very easy*	0-12.5<br>12.6-37.5<br>37.6-62.4<br>62.5-87.4<br>87.5-100	Sliding scale	Prim; Sec; FE	2021
		Compared to before the first lockdown, how easy have you found it to concentrate?	*Very difficult*<br>*Quite difficult*<br>*The same*<br>*Quite easy*<br>*Very easy*	0-12.5<br>12.6-37.5<br>37.6-62.4<br>62.5-87.4<br>87.5-100	Sliding scale	Prim; Sec; FE	2021
		Compared to before the first lockdown, how easy have you found it to think clearly?	*Very difficult*<br>*Quite difficult*<br>*The same*<br>*Quite easy*<br>*Very easy*	0-12.5<br>12.6-37.5<br>37.6-62.4<br>62.5-87.4<br>87.5-100	Sliding scale	Prim; Sec; FE	2021
		Would you take a COVID-19 vaccine (approved for use in the UK) if offered?	*Eager to get a COVID-19 vaccine*<br>*Willing to get a COVID-19 vaccine*	EAGER<br>WILLING	String	Prim; Sec; FE	2021

Note: A * in the *Matched* or *Year Included* columns indicates that the *Question* or *Label* column contain differences in the * indicated year or survey version

OxWell 2021 FE

		Not bothered about getting a COVID-19 vaccine	NOTBOTHERED
		Unwilling to get a COVID-19 vaccine	UNWILLING
		Anti-vaccination for COVID-19	ANTIVACCINATION
		Don't know	DONTKNOW

Note: A * in the *Matched* or *Year Included* columns indicates that the *Question* or *Label* column contain differences in the * indicated year or survey version

OxWell 2021 FE

BULLYING

CODE	Contingent	Question	Label	Value	Type	Matched	Year Included
		How well do you think your school/college deals with bullying	*Very badly*	0-12.5	Sliding scale	Prim; Sec; FE	2019; 2021
			Not very well	12.6-37.5			
			Average	37.6-62.4			
			Quite well	62.5-87.4			
			Extremely well	87.5-100			
		Have you been bullied in the last year?	*No*	0-12.5	Sliding scale	Prim*; Sec; FE	2019*; 2020; 2021
			Not often (e.g. once or twice)	12.6-37.5			
			Sometimes (e.g. monthly)	37.6-62.4			
			Quite often (e.g. weekly)	62.5-87.4			
			Most days	87.5-100			
		Is the bullying still happening?	Yes	Y	String	Prim; Sec; FE	2019; 2021
			No	N			
			Would rather not say	R			
		If you have been bullied in the last year in what way did it happen?			Tick box	Prim; Sec; FE	2019*; 2020; 2021
			Physical	Y			
			Verbal	Y			
			Cyber	Y			
			Isolated or Excluded	Y			
			Other	Y			
		What age is the bully or bullies?	In my year group	SAME	String	Sec: FE	2021
			In a younger year group	YOUNGER			
			In an older year group	OLDER			
			Both younger and older year groups	MIXED			
			An adult	ADULT			
			Would rather not say	R			
		If you have ever been bullied, did someone help you deal with it?			Tick box	Prim; Sec; FE	2019; 2021*
			Parent, step-parent or carer	Y			
			Someone else in your family	Y			
			Friend(s)	Y			
			A teacher or other adult at your school/college	Y			

Note: A * in the *Matched* or *Year Included* columns indicates that the *Question* or *Label* column contain differences in the * indicated year or survey version

OxWell 2021 FE

			A mental health worker (from outside the school/college) who visits the school/college	Y			
			A doctor, nurse (outside school/college), psychologist	Y			
			A counsellor, youth worker or social worker	Y			
			Internet advice sites, forums, chatrooms etc	Y			
			No-one	Y			
		Where does the bullying usually happen?	The journey to/from school/college	TOFROMSCHOOL	String	Prim; Sec; FE	2019; 2021*
			Online/social media sites	INTERNET			
			During lessons	CLASSROOM			
			In school/college but outside of lessons	OTHERSCHOL			
			At home	HOME			
			The place you are living now	PLACEMENT			
			Outside	OUTSIDE			
			Other	OTHER			
		What do students in your school/college do if they see someone being bullied?			Tick box	Prim; Sec; FE	2021
			Try to stop the bullying	Y			
			Tell a teacher/lecturer	Y			
			Laugh	Y			
			Join in	Y			
			Nothing	Y			

SELF-HARM

CODE	Contingent	Question	Label	Value	Type	Matched	Year Included
		**Have you ever deliberately self-harmed (for example by taking an overdose or deliberately injuring yourself in some other way)?**	*Yes*	Y	String	Sec; FE	2019*, 2020, 2021*
			No	N			
			Prefer not to say	R			
			Not sure what this means	D			
		**Have you ever deliberately injured yourself in some way?**	*Never*	0-12.5	Sliding scale	Sec; FE	2019, 2020, 2021
			Once or twice	12.6-37.5			
			A few times	37.6-62.4			

Note: A * in the *Matched* or *Year Included* columns indicates that the *Question* or *Label* column contain differences in the * indicated year or survey version

OxWell 2021 FE

		Weekly<br>*Daily*	62.5-87.4<br>87.5-100			
	**How old were you when you first self-harmed?**	*Age in years*	5 - 16	Numeric	Sec; FE	2019, 2020, 2021
	**When did you last self-harm?**	*In the last week*<br>*In the last month*<br>*in the past 3-6 months*<br>*6 months to a year ago*<br>*Over a year ago*	0-12.5<br>12.6-37.5<br>37.6-62.4<br>62.5-87.4<br>87.5-100	Sliding scale	Sec; FE	2019, 2020, 2021
	**How old were you when you last self-harmed?**	*Age in years*	5 - 16	Numeric	Sec; FE	2019, 2020, 2021
	**Have you ever deliberately taken an overdose (e.g. of pills or other medication)?**	*No*<br>*Yes - Once*<br>*Yes - On more than one occasion*	N<br>YONCE<br>YMORE	String	Sec; FE	2019, 2020, 2021*
	**How old were you when you first overdosed?**	*Age in years*	5 - 16	Numeric	Sec; FE	2019, 2020, 2021
	**When did you last take an overdose?**	*In the last week*<br>*In the last month*<br>*In the past 3-6 months*<br>*6 months to a year ago*<br>*Over a year ago*	0-12.5<br>12.6-37.5<br>37.6-62.4<br>62.5-87.4<br>87.5-100	Sliding scale	Sec; FE	2019, 2020, 2021
	**The last time this happened, in what way did you self-harm/ overdose?**	*Free text box*		Free text	Sec; FE	2019, 2020, 2021
	**Have you ever needed any medical treatment for your self-harm injury/overdose from… (Tick all that apply)**			Tick box	Sec; FE	2019, 2020, 2021
		My own first-aid	Y			
		Family-provided first-aid	Y			
		School/college nurse/first-aid at school/college	Y			
		Friends helped me	Y			
		GP (family doctor)	Y			
		Ambulance/paramedics	Y			
		Hospital A&E / acute mental health provision	Y			
		Hospital with overnight stay on ward	Y			

Note: A * in the *Matched* or *Year Included* columns indicates that the *Question* or *Label* column contain differences in the * indicated year or survey version

OxWell 2021 FE

		Other	Y			
	**Is there any support you would have liked to have accessed, or people you wish you could have talked to?**			Tick box	Sec; FE	2021
		Parent, step-parent or carer	Y			
		Brother or sister	Y			
		Someone else in your family	Y			
		Friend(s)	Y			
		GP (family doctor)	Y			
		Social Worker	Y			
		School/college Nurse/welfare staff	Y			
		Another adult at school/college	Y			
		A peer mentor at school/college	Y			
		CAMHS	Y			
		Support service given by charity	Y			
		An adult outside of school/college (at a sport club, another parent, family friend)	Y			
		A telephone/text help-line	Y			
		Website or online forum	Y			
		Other	Y			
		Please enter:		Free text box		
		None of these	Y			
	**Is there anything that prevented you from accessing support for your self-harm?**			Tick box	Sec; FE	2019, 2020
		Didn't know who to ask	Y			
		Didn't like the person providing support	Y			
		Didn't feel safe to share	Y			
		Worried I might not get taken seriously	Y			
		Didn't like to talk to strangers	Y			

Note: A * in the *Matched* or *Year Included* columns indicates that the *Question* or *Label* column contain differences in the * indicated year or survey version

OxWell 2021 FE

		Didn't want parents to know	Y		
		Didn't want other young people to know	Y		
		Didn't want teachers or staff in school/college to know	Y		
		Worried I would not be seen as a priority by the service	Y		
		Might have to wait too long to get help	Y		
		Too much hassle to get the help	Y		
		Did not want help	Y		
		Didn't want to burden anyone else	Y		
		Didn't want the stigma	Y		
		Didn't know where to get help	Y		
		Scared/worried about what people might say	Y		
		Other	Y		
	**Do any of the following reasons explain your motivation to take an overdose or harm yourself in some other way? (The following question refers to the LAST TIME you took an overdose or tried to harm yourself)**			Tick box	2021
		I wanted to show how desperate I was feeling	Y		
		I wanted to die	Y		
		I wanted to punish myself	Y		
		I wanted to frighten someone	Y		
		I wanted to get my own back on someone	Y		
		I wanted to get relief from a terrible state of mind	Y		
		I wanted to find out whether someone really loved me	Y		
		I wanted to get some attention	Y		
		I am not sure why I did it	Y		
	**Have you ever asked for support for your self-harm from the following?**				

Note: A * in the *Matched* or *Year Included* columns indicates that the *Question* or *Label* column contain differences in the * indicated year or survey version

OxWell 2021 FE

	**Parent, step-parent or carer**		Y			
	**Regarding this support, are you**	*Currently being offered support*	CURRENTSUPPORT	String	Sec; Fe	2021
		Previously been offered support	PREVSUPPORT			
		Not been offered support/been turned away	NOSUPPORT			
		Changed mind before getting the support	CHANGEDMIND			
	**Regarding this support, was it helpful?**	*Not helpful at all*	0-12.5	Sliding scale	Sec; Fe	2021
		Not helpful enough	12.6-37.5			
		Just about helpful enough	37.6-62.4			
		Quite helpful	62.5-87.4			
		Very helpful	87.5-100			
	**Brother or sister**		Y			
	**Regarding this support, are you**	*Currently being offered support*	CURRENTSUPPORT	String	Sec; Fe	2021
		Previously been offered support	PREVSUPPORT			
		Not been offered support/been turned away	NOSUPPORT			
		Changed mind before getting the support	CHANGEDMIND			
	**Regarding this support, was it helpful?**	*Not helpful at all*	0-12.5	Sliding scale	Sec; Fe	2021
		Not helpful enough	12.6-37.5			
		Just about helpful enough	37.6-62.4			
		Quite helpful	62.5-87.4			
		Very helpful	87.5-100			
	**Someone else in your family**		Y			
	**Regarding th CURRENTSUPPORT PREVSUPPORT NOSUPPORT CHANGEDMIND**			String	Sec; Fe	2021
		Previously been offered support	PREVSUPPORT			
		Not been offered support/been turned away	NOSUPPORT			
		Changed mind before getting the support	CHANGEDMIND			
	**Regarding this support, was it helpful?**	*Not helpful at all*	0-12.5	Sliding scale	Sec; Fe	2021
		Not helpful enough	12.6-37.5			
		Just about helpful enough	37.6-62.4			
		Quite helpful	62.5-87.4			

Note: A * in the *Matched* or *Year Included* columns indicates that the *Question* or *Label* column contain differences in the * indicated year or survey version

OxWell 2021 FE

		Very helpful	87.5-100			
	**Friend(s)**					
	**Regarding this support, are you**	*Currently being offered support*	CURRENTSUPPORT	String	Sec; Fe	2021
		Previously been offered support	PREVSUPPORT			
		Not been offered support/been turned away	NOSUPPORT			
		Changed mind before getting the support	CHANGEDMIND			
	**Regarding this support, was it helpful?**	*Not helpful at all*	0-12.5	Sliding scale	Sec; Fe	2021
		Not helpful enough	12.6-37.5			
		Just about helpful enough	37.6-62.4			
		Quite helpful	62.5-87.4			
		Very helpful	87.5-100			
	**GP (family doctor)**		Y			
	**Regarding this support, are you**	*Currently being offered support*	CURRENTSUPPORT	String	Sec; Fe	2021
		Previously been offered support	PREVSUPPORT			
		Not been offered support/been turned away	NOSUPPORT			
		Changed mind before getting the support	CHANGEDMIND			
	**Regarding this support, was it helpful?**	*Not helpful at all*	0-12.5	Sliding scale	Sec; Fe	2021
		Not helpful enough	12.6-37.5			
		Just about helpful enough	37.6-62.4			
		Quite helpful	62.5-87.4			
		Very helpful	87.5-100			
	**Social Worker**		Y			
	**Regarding this support, are you**	*Currently being offered support*	CURRENTSUPPORT	String	Sec; Fe	2021
		Previously been offered support	PREVSUPPORT			
		Not been offered support/been turned away	NOSUPPORT			
		Changed mind before getting the support	CHANGEDMIND			
	**Regarding this support, was it helpful?**	*Not helpful at all*	0-12.5	Sliding scale	Sec; Fe	2021
		Not helpful enough	12.6-37.5			
		Just about helpful enough	37.6-62.4			
		Quite helpful	62.5-87.4			

Note: A * in the *Matched* or *Year Included* columns indicates that the *Question* or *Label* column contain differences in the * indicated year or survey version

OxWell 2021 FE

		Very helpful	87.5-100			
	**School/college Nurse/Counsellor/Educational Mental health practitioner/other pastoral staff at school/college**		Y			
	**Regarding this support, are you**	*Currently being offered support*	CURRENTSUPPORT	String	Sec; Fe	2021
		Previously been offered support	PREVSUPPORT			
		Not been offered support/been turned away	NOSUPPORT			
		Changed mind before getting the support	CHANGEDMIND			
	**Regarding this support, was it helpful?**	*Not helpful at all*	0-12.5	Sliding scale	Sec; Fe	2021
		Not helpful enough	12.6-37.5			
		Just about helpful enough	37.6-62.4			
		Quite helpful	62.5-87.4			
		Very helpful	87.5-100			
	**Another adult at school/college**		Y			
	**Regarding this support, are you**	*Currently being offered support*	CURRENTSUPPORT	String	Sec; Fe	2021
		Previously been offered support	PREVSUPPORT			
		Not been offered support/been turned away	NOSUPPORT			
		Changed mind before getting the support	CHANGEDMIND			
	**Regarding this support, was it helpful?**	*Not helpful at all*	0-12.5	Sliding scale	Sec; Fe	2021
		Not helpful enough	12.6-37.5			
		Just about helpful enough	37.6-62.4			
		Quite helpful	62.5-87.4			
		Very helpful	87.5-100			
	**A peer mentor at school/college**		Y			
	**Regarding this support, are you**	*Currently being offered support*	CURRENTSUPPORT	String	Sec; Fe	2021
		Previously been offered support	PREVSUPPORT			
		Not been offered support/been turned away	NOSUPPORT			
		Changed mind before getting the support	CHANGEDMIND			
	**Regarding this support, was it helpful?**	*Not helpful at all*	0-12.5	Sliding scale	Sec; Fe	2021
		Not helpful enough	12.6-37.5			
		Just about helpful enough	37.6-62.4			

Note: A * in the *Matched* or *Year Included* columns indicates that the *Question* or *Label* column contain differences in the * indicated year or survey version

OxWell 2021 FE

		Quite helpful<br>*Very helpful*	62.5-87.4<br>87.5-100			
	**CAMHS (NHS Child and adolescent Â mental health services)**		Y			
	**Regarding this support, are you**	*Currently being offered support*<br>*Previously been offered support*<br>*Not been offered support/been turned away*<br>*Changed mind before getting the support*	CURRENTSUPPORT<br>PREVSUPPORT<br>NOSUPPORT<br>CHANGEDMIND	String	Sec; Fe	2021
	**Regarding this support, was it helpful?**	*Not helpful at all*<br>*Not helpful enough*<br>*Just about helpful enough*<br>*Quite helpful*<br>*Very helpful*	0-12.5<br>12.6-37.5<br>37.6-62.4<br>62.5-87.4<br>87.5-100	Sliding scale	Sec; Fe	2021
	**Support service given by charity**		Y			
	**Regarding this support, are you**	*Currently being offered support*<br>*Previously been offered support*<br>*Not been offered support/been turned away*<br>*Changed mind before getting the support*	CURRENTSUPPORT<br>PREVSUPPORT<br>NOSUPPORT<br>CHANGEDMIND	String	Sec; Fe	2021
	**Regarding this support, was it helpful?**	*Not helpful at all*<br>*Not helpful enough*<br>*Just about helpful enough*<br>*Quite helpful*<br>*Very helpful*	0-12.5<br>12.6-37.5<br>37.6-62.4<br>62.5-87.4<br>87.5-100	Sliding scale	Sec; Fe	2021
	**An adult outside of school/college (at a sport club, another parent, family friend)**		Y			
	**Regarding this support, are you**	*Currently being offered support*<br>*Previously been offered support*<br>*Not been offered support/been turned away*<br>*Changed mind before getting the support*	CURRENTSUPPORT<br>PREVSUPPORT<br>NOSUPPORT<br>CHANGEDMIND	String	Sec; Fe	2021
	**Regarding this support, was it helpful?**	*Not helpful at all*<br>*Not helpful enough*	0-12.5<br>12.6-37.5	Sliding scale	Sec; Fe	2021

Note: A * in the *Matched* or *Year Included* columns indicates that the *Question* or *Label* column contain differences in the * indicated year or survey version

OxWell 2021 FE

			Just about helpful enough	37.6-62.4			
			Quite helpful	62.5-87.4			
			Very helpful	87.5-100			
		**A telephone/text helpline**					
		**Regarding this support, are you**	*Currently being offered support*	CURRENTS UPPORT	String	Sec; Fe	2021
			Previously been offered support	PREVSUPP ORT			
			Not been offered support/been turned away	NOSUPPOR T			
			Changed mind before getting the support	CHANGEDMI ND			
		**Regarding this support, was it helpful?**	*Not helpful at all*	0-12.5	Sliding scale	Sec; Fe	2021
			Not helpful enough	12.6-37.5			
			Just about helpful enough	37.6-62.4			
			Quite helpful	62.5-87.4			
			Very helpful	87.5-100			
		**Website or online forum**		Y			
		**Regarding this support, are you**	*Currently being offered support*	CURRENTS UPPORT	String	Sec; Fe	2021
			Previously been offered support	PREVSUPP ORT			
			Not been offered support/been turned away	NOSUPPOR T			
			Changed mind before getting the support	CHANGEDMI ND			
		**Regarding this support, was it helpful?**	*Not helpful at all*	0-12.5	Sliding scale	Sec; Fe	2021
			Not helpful enough	12.6-37.5			
			Just about helpful enough	37.6-62.4			
			Quite helpful	62.5-87.4			
			Very helpful	87.5-100			
		**Other**					
		**Please enter**		Y	Free text box		
		**Regarding this support, are you**	*Currently being offered support*	CURRENTS UPPORT	String	Sec; Fe	2021
			Previously been offered support	PREVSUPP ORT			
			Not been offered support/been turned away	NOSUPPOR T			
			Changed mind before getting the support	CHANGEDMI ND			
		**Regarding this support, was it helpful?**	*Not helpful at all*	0-12.5	Sliding scale	Sec; Fe	2021

Note: A * in the *Matched* or *Year Included* columns indicates that the *Question* or *Label* column contain differences in the * indicated year or survey version

OxWell 2021 FE

		Not helpful enough<br>*Just about helpful enough*<br>*Quite helpful*<br>*Very helpful*	12.6-37.5<br>37.6-62.4<br><br>62.5-87.4<br>87.5-100			
	**None of these**		Y			
	**Have you ever come across any content related to self-harm online?**	*Never*<br>*Once or twice*<br>*A few times*<br>*Weekly*<br>*Daily*	0-12.5<br>12.6-37.5<br>37.6-62.4<br>62.5-87.4<br>87.5-100	String	Sec; FE	2019, 2020, 2021
	**Have you ever seriously thought about taking an overdose or trying to harm yourself but not actually done so?**	*No*<br>*Yes – last time was within the past month*<br>*Yes – last time was within the past year*<br>*Yes – last time was more than a year ago*	NO<br>WITHINMNTH<br>WITHINYR<br>GTYR	String	Sec; FE	2019,2021
	**Have you ever told someone you were going to harm or kill yourself?**	*Never*<br>*Rarely*<br>*Occasionally*<br>*Frequently*<br>*Always*	0-12.5<br>12.6-37.5<br>37.6-62.4<br>62.5-87.4<br>87.5-100	Sliding scale	Sec; FE	2019, 2020, 2021
	**Has anyone among your family or close friends died by suicide?**	*No*<br>*Yes – in the past 12 months*<br>*Yes – more than a year ago*	N<br>YLAST12<br>YGTYR	String	Sec; FE	2019, 2020, 2021*
	**Has anyone among your family attempted suicide or tried to harm themselves?**	*No*<br>*Yes – in the past 12 months*<br>*Yes – more than a year ago*	N<br>YLAST12<br>YGTYR	String	Sec; FE	2019, 2020, 2021*
	**Has anyone among your close friends attempted suicide or tried to harm themselves?**	*No*<br>*Yes – in the past 12 months*<br>*Yes – more than a year ago*	N<br>YLAST12<br>YGTYR	String	Sec; FE	2019, 2020, 2021*

Note: A * in the *Matched* or *Year Included* columns indicates that the *Question* or *Label* column contain differences in the * indicated year or survey version

OxWell 2021 FE

MENTAL HEALTH

CODE	Contingent	Question	Label	Value	Type	Matched	Year Included
		I've been feeling optimistic about the future	*None of the time*	0-12.5	Sliding scale	Prim*; Sec; FE	2019; 2020; 2021
			Rarely	12.6-37.5			
			Some of the time	37.6-62.4			
			Often	62.5-87.4			
			All of the time	87.5-100			
		I've been feeling useful	*None of the time*	0-12.5	Sliding scale	Prim; Sec; FE	2019; 2020; 2021
			Rarely	12.6-37.5			
			Some of the time	37.6-62.4			
			Often	62.5-87.4			
			All of the time	87.5-100			
		I've been feeling relaxed	*None of the time*	0-12.5	Sliding scale	Prim; Sec; FE	2019; 2020; 2021
			Rarely	12.6-37.5			
			Some of the time	37.6-62.4			
			Often	62.5-87.4			
			All of the time	87.5-100			
		I've been feeling interested in other people	*None of the time*	0-12.5	Sliding scale	Prim; Sec; FE	2019; 2020; 2021
			Rarely	12.6-37.5			
			Some of the time	37.6-62.4			
			Often	62.5-87.4			
			All of the time	87.5-100			
		I've had energy to spare	*None of the time*	0-12.5	Sliding scale	Prim; Sec; FE	2019; 2020; 2021
			Rarely	12.6-37.5			
			Some of the time	37.6-62.4			
			Often	62.5-87.4			
			All of the time	87.5-100			
		I've been dealing with problems well	*None of the time*	0-12.5	Sliding scale	Prim; Sec; FE	2019; 2020; 2021
			Rarely	12.6-37.5			
			Some of the time	37.6-62.4			
			Often	62.5-87.4			
			All of the time	87.5-100			
		I've been thinking clearly	*None of the time*	0-12.5	Sliding scale	Prim; Sec; FE	2019; 2020; 2021
			Rarely	12.6-37.5			
			Some of the time	37.6-62.4			

Note: A * in the *Matched* or *Year Included* columns indicates that the *Question* or *Label* column contain differences in the * indicated year or survey version

OxWell 2021 FE

		Often	62.5-87.4			
		All of the time	87.5-100			
	I've been feeling good about myself	*None of the time*	0-12.5	Sliding scale	Prim; Sec; FE	2019; 2020; 2021
		Rarely	12.6-37.5			
		Some of the time	37.6-62.4			
		Often	62.5-87.4			
		All of the time	87.5-100			
	I've been feeling confident	*None of the time*	0-12.5	Sliding scale	Prim; Sec; FE	2019; 2020; 2021
		Rarely	12.6-37.5			
		Some of the time	37.6-62.4			
		Often	62.5-87.4			
		All of the time	87.5-100			
	I've been able to make my own mind up about things	*None of the time*	0-12.5	Sliding scale	Prim; Sec; FE	2019; 2020; 2021
		Rarely	12.6-37.5			
		Some of the time	37.6-62.4			
		Often	62.5-87.4			
		All of the time	87.5-100			
	I've been feeling loved	*None of the time*	0-12.5	Sliding scale	Prim; Sec; FE	2019; 2020; 2021
		Rarely	12.6-37.5			
		Some of the time	37.6-62.4			
		Often	62.5-87.4			
		All of the time	87.5-100			
	I've been interested in new things	*None of the time*	0-12.5	Sliding scale	Prim; Sec; FE	2019; 2020; 2021
		Rarely	12.6-37.5			
		Some of the time	37.6-62.4			
		Often	62.5-87.4			
		All of the time	87.5-100			
	I've been feeling cheerful	*None of the time*	0-12.5	Sliding scale	Prim; Sec; FE	2019; 2020; 2021
		Rarely	12.6-37.5			
		Some of the time	37.6-62.4			
		Often	62.5-87.4			
		All of the time	87.5-100			
	I've been feeling close to other people	*None of the time*	0-12.5	Sliding scale	Prim; Sec; FE	2019; 2020; 2021
		Rarely	12.6-37.5			
		Some of the time	37.6-62.4			

Note: A * in the *Matched* or *Year Included* columns indicates that the *Question* or *Label* column contain differences in the * indicated year or survey version

OxWell 2021 FE

		Often<br>*All of the time*	62.5-87.4<br>87.5-100			
	I feel sad or empty	*Never*<br>*Sometimes*<br>*Often*<br>*Always*	NEVER<br>SOMETIMES<br>OFTEN<br>ALWAYS	String	Sec; FE	2020; 2021
	I worry when I think I have done poorly at something	*Never*<br>*Sometimes*<br>*Often*<br>*Always*	NEVER<br>SOMETIMES<br>OFTEN<br>ALWAYS	String	Sec; FE	2020; 2021
	I would feel afraid of being on my own at home	*Never*<br>*Sometimes*<br>*Often*<br>*Always*	NEVER<br>SOMETIMES<br>OFTEN<br>ALWAYS	String	Sec; FE	2020; 2021
	Nothing is much fun anymore	*Never*<br>*Sometimes*<br>*Often*<br>*Always*	NEVER<br>SOMETIMES<br>OFTEN<br>ALWAYS	String	Sec; FE	2020; 2021
	I worry that something awful will happen to someone in my family	*Never*<br>*Sometimes*<br>*Often*<br>*Always*	NEVER<br>SOMETIMES<br>OFTEN<br>ALWAYS	String	Sec; FE	2020; 2021
	I am afraid of being in crowded places (like shopping centres, the movies, buses, busy playgrounds)	*Never*<br>*Sometimes*<br>*Often*<br>*Always*	NEVER<br>SOMETIMES<br>OFTEN<br>ALWAYS	String	Sec; FE	2020; 2021
	I worry what other people think of me	*Never*<br>*Sometimes*<br>*Often*<br>*Always*	NEVER<br>SOMETIMES<br>OFTEN<br>ALWAYS	String	Sec; FE	2020; 2021
	I have trouble sleeping	*Never*<br>*Sometimes*<br>*Often*<br>*Always*	NEVER<br>SOMETIMES<br>OFTEN<br>ALWAYS	String	Sec; FE	2020; 2021

Note: A * in the *Matched* or *Year Included* columns indicates that the *Question* or *Label* column contain differences in the * indicated year or survey version

OxWell 2021 FE

	I feel scared if I have to sleep on my own	*Never*<br>*Sometimes*<br>*Often*<br>*Always*	NEVER<br>SOMETIMES<br>OFTEN<br>ALWAYS	String	Sec; FE	2020; 2021
	I have problems with my appetite	*Never*<br>*Sometimes*<br>*Often*<br>*Always*	NEVER<br>SOMETIMES<br>OFTEN<br>ALWAYS	String	Sec; FE	2020; 2021
	I suddenly become dizzy or faint when there is no reason for this	*Never*<br>*Sometimes*<br>*Often*<br>*Always*	NEVER<br>SOMETIMES<br>OFTEN<br>ALWAYS	String	Sec; FE	2020; 2021
	I have to do some things over and over again (like washing my hands, cleaning or putting things in a certain order)	*Never*<br>*Sometimes*<br>*Often*<br>*Always*	NEVER<br>SOMETIMES<br>OFTEN<br>ALWAYS	String	Sec; FE	2020; 2021
	I have no energy for things	*Never*<br>*Sometimes*<br>*Often*<br>*Always*	NEVER<br>SOMETIMES<br>OFTEN<br>ALWAYS	String	Sec; FE	2020; 2021
	I suddenly start to tremble or shake when there is no reason for this	*Never*<br>*Sometimes*<br>*Often*<br>*Always*	NEVER<br>SOMETIMES<br>OFTEN<br>ALWAYS	String	Sec; FE	2020; 2021
	I cannot think clearly	*Never*<br>*Sometimes*<br>*Often*<br>*Always*	NEVER<br>SOMETIMES<br>OFTEN<br>ALWAYS	String	Sec; FE	2020; 2021
	I feel worthless	*Never*<br>*Sometimes*<br>*Often*<br>*Always*	NEVER<br>SOMETIMES<br>OFTEN<br>ALWAYS	String	Sec; FE	2020; 2021
		Never	NEVER	String	Sec; FE	2020; 2021

Note: A * in the *Matched* or *Year Included* columns indicates that the *Question* or *Label* column contain differences in the * indicated year or survey version

OxWell 2021 FE

	I have to think of special thoughts (like numbers or words) to stop bad things from happening.	*Sometimes*<br>*Often*<br>*Always*	SOMETIMES<br>OFTEN<br>ALWAYS			
	I think about death	*Never*<br>*Sometimes*<br>*Often*<br>*Always*	NEVER<br>SOMETIMES<br>OFTEN<br>ALWAYS	String	Sec; FE	2020; 2021
	I feel like I don't want to move	*Never*<br>*Sometimes*<br>*Often*<br>*Always*	NEVER<br>SOMETIMES<br>OFTEN<br>ALWAYS	String	Sec; FE	2020; 2021
	I worry that I will suddenly get a scared feeling when there is nothing to be afraid of	*Never*<br>*Sometimes*<br>*Often*<br>*Always*	NEVER<br>SOMETIMES<br>OFTEN<br>ALWAYS	String	Sec; FE	2020; 2021
	I am tired a lot	*Never*<br>*Sometimes*<br>*Often*<br>*Always*	NEVER<br>SOMETIMES<br>OFTEN<br>ALWAYS	String	Sec; FE	2020; 2021
	I feel afraid that I will make a fool of myself in front of people	*Never*<br>*Sometimes*<br>*Often*<br>*Always*	NEVER<br>SOMETIMES<br>OFTEN<br>ALWAYS	String	Sec; FE	2020; 2021
	I have to do some things in just the right way to stop bad things from happening	*Never*<br>*Sometimes*<br>*Often*<br>*Always*	NEVER<br>SOMETIMES<br>OFTEN<br>ALWAYS	String	Sec; FE	2020; 2021
	I feel restless	*Never*<br>*Sometimes*<br>*Often*<br>*Always*	NEVER<br>SOMETIMES<br>OFTEN<br>ALWAYS	String	Sec; FE	2020; 2021
	I worry that something bad will happen to me	*Never*<br>*Sometimes*<br>*Often*	NEVER<br>SOMETIMES<br>OFTEN	String	Sec; FE	2020; 2021

Note: A * in the *Matched* or *Year Included* columns indicates that the *Question* or *Label* column contain differences in the * indicated year or survey version

OxWell 2021 FE

		Always	ALWAYS			
	Overall, are you basically satisfied with your life?	*Unsatisfied most of the time*	0-12.5	Sliding scale	Prim; Sec; FE	2019; 2020; 2021
		Quite unsatisfied	12.6-37.5			
		Neither satisfied nor unsatisfied	37.6-62.4			
		Quite satisfied	62.5-87.4			
		Satisfied	87.5-100			
	How often have you been so worried about something you can not sleep at night?	*Never*	0-12.5	Sliding scale	Prim; Sec; FE	2019; 2020; 2021
		Once or twice	12.6-37.5			
		Sometimes	37.6-62.4			
		Most nights	62.5-87.4			
		Every night	87.5-100			
	Overall, how confident about your future do you generally feel?	Not at all confident	0-12.5	Sliding scale	Prim ; Sec; FE	2019;2021
		Not very confident	12.6-37.5			
		Quite confident	37.6-62.4			
		Confident	62.5-87.4			
		Extremely confident	87.5-100			
	How proud do you feel of things you have achieved in your life?	Not at all	0-12.5	Sliding scale	Prim ; Sec; FE	2019; 2021
		Not very	12.6-37.5			
		A bit	37.6-62.4			
		Quite	62.5-87.4			
		Extremely	87.5-100			

Note: A * in the *Matched* or *Year Included* columns indicates that the *Question* or *Label* column contain differences in the * indicated year or survey version

OxWell 2021 FE

ADVERSE CHILDHOOD EXPERIENCES

CODE	Contingent	Question	Label	Value	Type	Matched	Year Included
		Many young people experience stressful life events that can affect their health and wellbeing<br><br>***Please read the 10 statements below. Count the number of statements that apply to you and select that number in the drop down box.***<br><br>***At any point since you were born...***<br><br>***1. Your parents or guardians were separated or divorced***<br>***2. You lived with a household member who served time in jail or prison***<br>***3. You lived with a household member who was depressed, mentally unwell or attempted suicide***<br>***4. You saw or heard household members hurt or threaten to hurt each other***<br>***5. A household member swore at, insulted, humiliated, or put you down in a way that scared you OR a household member acted in a way that made you afraid that you might be physically hurt***<br>***6. Someone touched your private parts or asked you to touch their private parts in a sexual way that was unwanted, against your will, or made you feel uncomfortable***<br>***7. More than once, you went without food, clothing, a place to live, or had no one to protect you***<br>***8. Someone pushed, grabbed, slapped or threw something at you OR you were hit so hard that you were injured or had marks***<br>***9. You lived with someone who had a problem with drinking or using drugs***<br>***10. You often felt unsupported, unloved and/or unprotected***	*Number of experiences*	1 - 10	Numeric	FE	2019: 2020; 2021

Note: A * in the *Matched* or *Year Included* columns indicates that the *Question* or *Label* column contain differences in the * indicated year or survey version

OxWell 2021 FE

MENTAL HEALTH SERVICES

CODE	Contingent	Question	Label	Value	Type	Matched	Year Included
		Have you ever felt you had a mental health problem	*Yes*	Y	String	Sec; FE	2021
			No	N			
		Do you know who provides Mental Health support in your school/college (where to go when you are worried and want to talk to an adult)?	*Yes*	Y	String	Prim*; Sec; FE	2019*; 2020; 2021*
			No	N			
			Not sure	D			
		Who provides mental health support in your school/college?			Tick box	Prim*; Sec; FE	2021
			Education Mental Health Practitioner (EMHP)	Y			
			School/college nurse	Y			
			Other mental health workers who visit the school/college (for example, a charity)	Y			
			School/college counsellor/psychologist	Y			
			Class teacher/lecturer	Y			
			Head of year	Y			
			Teaching assistants	Y			
			SENCO	Y			
			Peer mentor	Y			
			Other adult in school/college	Y			
		Is it easy to access mental health support at school/college	*Very difficult*	0-12.5	Sliding scale	Prim*; Sec; FE	2020; 2021
			Quite difficult	12.6-37.5			
			Sometimes difficult	37.6-62.4			
			Quite easy	62.5-87.4			
			Very easy	87.5-100			
		**Have you ever tried to ask for support for a mental health problem from the following:**					
		**Parent, step-parent or carer**		Y			
		**Regarding this support, are you**	*Currently being offered support*	CURRENTSUPPORT	String	Sec; Fe	2021
			Previously been offered support	PREVSUPPORT			

Note: A * in the *Matched* or *Year Included* columns indicates that the *Question* or *Label* column contain differences in the * indicated year or survey version

OxWell 2021 FE

		Not been offered support/been turned away	NOSUPPORT			
		Changed mind before getting the support	CHANGEDMIND			
	**Regarding this support, was it helpful?**	*Not helpful at all*	0-12.5	Sliding scale	Sec; Fe	2021
		Not helpful enough	12.6-37.5			
		Just about helpful enough	37.6-62.4			
		Quite helpful	62.5-87.4			
		Very helpful	87.5-100			
	**Brother or sister**		Y			
	**Regarding th CURRENTSUPPORT PREVSUPPORT NOSUPPORT CHANGEDMIND**			String	Sec; Fe	2021
		Previously been offered support	PREVSUPPORT			
		Not been offered support/been turned away	NOSUPPORT			
		Changed mind before getting the support	CHANGEDMIND			
	**Regarding this support, was it helpful?**	*Not helpful at all*	0-12.5	Sliding scale	Sec; Fe	2021
		Not helpful enough	12.6-37.5			
		Just about helpful enough	37.6-62.4			
		Quite helpful	62.5-87.4			
		Very helpful	87.5-100			
	**Someone else in your family**		Y			
	**Regarding this support, are you**	*Currently being offered support*	CURRENTSUPPORT	String	Sec; Fe	2021
		Previously been offered support	PREVSUPPORT			
		Not been offered support/been turned away	NOSUPPORT			
		Changed mind before getting the support	CHANGEDMIND			
	**Regarding this support, was it helpful?**	*Not helpful at all*	0-12.5	Sliding scale	Sec; Fe	2021
		Not helpful enough	12.6-37.5			
		Just about helpful enough	37.6-62.4			
		Quite helpful	62.5-87.4			
		Very helpful	87.5-100			

Note: A * in the *Matched* or *Year Included* columns indicates that the *Question* or *Label* column contain differences in the * indicated year or survey version

OxWell 2021 FE

	**Friend(s)**		Y			
	**Regarding th CURRENTSUPPORT PREVSUPPORT NOSUPPORT CHANGEDMIND**			String	Sec; Fe	2021
		Previously been offered support	PREVSUPPORT			
		Not been offered support/been turned away	NOSUPPORT			
		Changed mind before getting the support	CHANGEDMIND			
	**Regarding this support, was it helpful?**	*Not helpful at all*	0-12.5	Sliding scale	Sec; Fe	2021
		Not helpful enough	12.6-37.5			
		Just about helpful enough	37.6-62.4			
		Quite helpful	62.5-87.4			
		Very helpful	87.5-100			
	**GP (family doctor)**		Y			
	**Regarding this support, are you**	*Currently being offered support*		String	Sec; Fe	2021
		Previously been offered support				
		Not been offered support/been turned away	CURRENTSUPPORT			
		Changed mind before getting the support	PREVSUPPORT			
	**Regarding this support, was it helpful?**	*Not helpful at all*	NOSUPPORT	Sliding scale	Sec; Fe	2021
		Not helpful enough	CHANGEDMIND			
		Just about helpful enough	37.6-62.4			
		Quite helpful	62.5-87.4			
		Very helpful	87.5-100			
	**Social Worker**		Y			
	**Regarding this support, are you**	*Currently being offered support*	CURRENTSUPPORT	String	Sec; Fe	2021
		Previously been offered support	PREVSUPPORT			
		Not been offered support/been turned away	NOSUPPORT			
		Changed mind before getting the support	CHANGEDMIND			

Note: A * in the *Matched* or *Year Included* columns indicates that the *Question* or *Label* column contain differences in the * indicated year or survey version

OxWell 2021 FE

	**Regarding this support, was it helpful?**	*Not helpful at all*	0-12.5	Sliding scale	Sec; Fe	2021
		Not helpful enough	12.6-37.5			
		Just about helpful enough	37.6-62.4			
		Quite helpful	62.5-87.4			
		Very helpful	87.5-100			
	**School/college Nurse/Counsellor/Educational Mental health practitioner/other pastoral staff at school/college**		Y			
	**Regarding this support, are you**	*Currently being offered support*	CURRENTSUPPORT	String	Sec; Fe	2021
		Previously been offered support	PREVSUPPORT			
		Not been offered support/been turned away	NOSUPPORT			
		Changed mind before getting the support	CHANGEDMIND			
	**Regarding this support, was it helpful?**	*Not helpful at all*	0-12.5	Sliding scale	Sec; Fe	2021
		Not helpful enough	12.6-37.5			
		Just about helpful enough	37.6-62.4			
		Quite helpful	62.5-87.4			
		Very helpful	87.5-100			
	**Another adult at school/college**		Y			
	**Regarding this support, are you**	*Currently being offered support*	CURRENTSUPPORT	String	Sec; Fe	2021
		Previously been offered support	PREVSUPPORT			
		Not been offered support/been turned away	NOSUPPORT			
		Changed mind before getting the support	CHANGEDMIND			
	**Regarding this support, was it helpful?**	*Not helpful at all*	0-12.5	Sliding scale	Sec; Fe	2021
		Not helpful enough	12.6-37.5			
		Just about helpful enough	37.6-62.4			
		Quite helpful	62.5-87.4			
		Very helpful	87.5-100			
	**A peer mentor at school/college**		Y			
	**Regarding this support, are you**	*Currently being offered support*	CURRENTSUPPORT	String	Sec; Fe	2021
		Previously been offered support	PREVSUPPORT			

Note: A * in the *Matched* or *Year Included* columns indicates that the *Question* or *Label* column contain differences in the * indicated year or survey version

OxWell 2021 FE

		Not been offered support/been turned away	NOSUPPORT			
		Changed mind before getting the support	CHANGEDMIND			
	**Regarding this support, was it helpful?**	*Not helpful at all*	0-12.5	Sliding scale	Sec; Fe	2021
		Not helpful enough	12.6-37.5			
		Just about helpful enough	37.6-62.4			
		Quite helpful	62.5-87.4			
		Very helpful	87.5-100			
	**CAMHS (NHS Child and adolescent Â mental health services)**		Y			
	**Regarding this support, are you**	*Currently being offered support*	CURRENTSUPPORT	String	Sec; Fe	2021
		Previously been offered support	PREVSUPPORT			
		Not been offered support/been turned away	NOSUPPORT			
		Changed mind before getting the support	CHANGEDMIND			
	**Regarding this support, was it helpful?**	*Not helpful at all*	0-12.5	Sliding scale	Sec; Fe	2021
		Not helpful enough	12.6-37.5			
		Just about helpful enough	37.6-62.4			
		Quite helpful	62.5-87.4			
		Very helpful	87.5-100			
	**Support service given by charity**		Y			
	**Regarding this support, are you**	*Currently being offered support*	CURRENTSUPPORT	String	Sec; Fe	2021
		Previously been offered support	PREVSUPPORT			
		Not been offered support/been turned away	NOSUPPORT			
		Changed mind before getting the support	CHANGEDMIND			
	**Regarding this support, was it helpful?**	*Not helpful at all*	0-12.5	Sliding scale	Sec; Fe	2021
		Not helpful enough	12.6-37.5			
		Just about helpful enough	37.6-62.4			
		Quite helpful	62.5-87.4			
		Very helpful	87.5-100			

Note: A * in the *Matched* or *Year Included* columns indicates that the *Question* or *Label* column contain differences in the * indicated year or survey version

OxWell 2021 FE

	**An adult outside of school/college (at a sport club, another parent, family friend)**		Y			
	**Regarding this support, are you**	*Currently being offered support*	CURRENTSUPPORT	String	Sec; Fe	2021
		Previously been offered support	PREVSUPPORT			
		Not been offered support/been turned away	NOSUPPORT			
		Changed mind before getting the support	CHANGEDMIND			
	**Regarding this support, was it helpful?**	*Not helpful at all*	0-12.5	Sliding scale	Sec; Fe	2021
		Not helpful enough	12.6-37.5			
		Just about helpful enough	37.6-62.4			
		Quite helpful	62.5-87.4			
		Very helpful	87.5-100			
	**A telephone/text helpline**		Y			
	**Regarding this support, are you**	*Currently being offered support*	CURRENTSUPPORT	String	Sec; Fe	2021
		Previously been offered support	PREVSUPPORT			
		Not been offered support/been turned away	NOSUPPORT			
		Changed mind before getting the support	CHANGEDMIND			
	**Regarding this support, was it helpful?**	*Not helpful at all*	0-12.5	Sliding scale	Sec; Fe	2021
		Not helpful enough	12.6-37.5			
		Just about helpful enough	37.6-62.4			
		Quite helpful	62.5-87.4			
		Very helpful	87.5-100			
	**Website or online forum**		Y			
	**Regarding this support, are you**	*Currently being offered support*	CURRENTSUPPORT	String	Sec; Fe	2021
		Previously been offered support	PREVSUPPORT			
		Not been offered support/been turned away	NOSUPPORT			
		Changed mind before getting the support	CHANGEDMIND			
	**Regarding this support, was it helpful?**	*Not helpful at all*	0-12.5	Sliding scale	Sec; Fe	2021
		Not helpful enough	12.6-37.5			

Note: A * in the *Matched* or *Year Included* columns indicates that the *Question* or *Label* column contain differences in the * indicated year or survey version

OxWell 2021 FE

		Just about helpful enough	37.6-62.4			
		Quite helpful	62.5-87.4			
		Very helpful	87.5-100			
	**Other**					
	**Please enter**		Y	Free text box		
	**Regarding this support, are you**	*Currently being offered support*	CURRENTSUPPORT	String	Sec; Fe	2021
		Previously been offered support	PREVSUPPORT			
		Not been offered support/been turned away	NOSUPPORT			
		Changed mind before getting the support	CHANGEDMIND			
	**Regarding this support, was it helpful?**	*Not helpful at all*	0-12.5	Sliding scale	Sec; Fe	2021
		Not helpful enough	12.6-37.5			
		Just about helpful enough	37.6-62.4			
		Quite helpful	62.5-87.4			
		Very helpful	87.5-100			
	**None of these**		Y			
	Is there any support you would have liked to have accessed, or people you wish you could have talked to?			Tick box	Sec; FE	2021
		Parent, step-parent or carer	Y			
		Brother or sister	Y			
		Someone else in your family	Y			
		Friend(s)	Y			
		GP (family doctor)	Y			
		Social Worker	Y			
		School/college Nurse/Counsellor/Educational Mental health practitioner/SENCO/other pastoral staff at school/college	Y			
		A peer mentor at school/college	Y			
		CAMHS (NHS Child and adolescent mental health services)	Y			
		Support Service given by a charity	Y			

Note: A * in the *Matched* or *Year Included* columns indicates that the *Question* or *Label* column contain differences in the * indicated year or survey version

OxWell 2021 FE

		An adult outside of school/college (at a sport club, another parent, family friend)	Y			
		A telephone/text help-line	Y			
		Website or online forum	Y			
		Other	Y			
		Please enter:	Y			
		None of these	Y			
	Is there anything that prevented you from accessing support?			Tick box	Prim*; Sec; FE	2020; 2021*
		Didn't know who to ask	Y			
		Didn't like the person providing support	Y			
		Didn't feel safe to share	Y			
		Worried I might not get taken seriously	Y			
		Didn't like to talk to strangers	Y			
		Didn't want parents to know	Y			
		Didn't want other young people to know	Y			
		Didn't want teachers or staff in school/college to know	Y			
		Worried I would not be seen as a priority by the service	Y			
		Might have to wait too long to get help	Y			
		Too much hassle to get the help	Y			
		Did not want help	Y			
		Didn't want to burden anyone else	Y			
		Didn't want the stigma	Y			
		Scared/worried about what people might say	Y			
		Other	Y			
		Please enter:		Free text box		

Note: A * in the *Matched* or *Year Included* columns indicates that the *Question* or *Label* column contain differences in the * indicated year or survey version

OxWell 2021 FE

RELATIONSHIPS

CODE	Contingent	Question	Label	Value	Type	Matched	Year Included
		How easy do you find it to make and keep friends?	*Very difficult*<br>*Quite difficult*<br>*Sometimes difficult*<br>*Quite easy*<br>*Very easy*	0-12.5<br>12.6-37.5<br>37.6-62.4<br>62.5-87.4<br>87.5-100	String	Prim; Sec; FE	2019; 2021
		How well do you get along with other people in your household?	*Very well or well*<br>*Most of the time well*<br>*Not well or not at all well*	WELL<br>WELLMOST<br>NOTWELL	String	Prim; Sec; FE	2020; 2021
		How well do you get along with your friends?	*Very well or well*<br>*Most of the time well*<br>*Not well or not at all well*	WELL<br>WELLMOST<br>NOTWELL	String	Prim; Sec; FE	2020; 2021
		How often do you feel that you have no one to talk to (in person/online/phone)?	*Hardly ever or never*<br>*Some of the time*<br>*Often*	NEVER<br>SOMETIMES<br>OFTEN	String	Prim; Sec; FE	2020; 2021
		How often do you feel left out?	*Hardly ever or never*<br>*Some of the time*<br>*Often*	NEVER<br>SOMETIMES<br>OFTEN	String	Prim; Sec; FE	2020; 2021
		How often do you feel alone?	*Hardly ever or never*<br>*Some of the time*<br>*Often*	NEVER<br>SOMETIMES<br>OFTEN	String	Prim; Sec; FE	2021
		How often do you feel lonely?	*Hardly ever or never*<br>*Some of the time*<br>*Often*	NEVER<br>SOMETIMES<br>OFTEN	String	Prim; Sec; FE	2020; 2021

Note: A * in the *Matched* or *Year Included* columns indicates that the *Question* or *Label* column contain differences in the * indicated year or survey version

OxWell 2021 FE

SEXUAL HEALTH

CODE	Contingent	Question	Label	Value	Type	Matched	Year Included
		Do you wish to opt out of the RSE questions?	*Yes*	Y	String	Sec; FE	2019; 2021
			No	N			
		Have you ever had sex (sexual intercourse)?	*Yes*	Y	String	FE	2019
			No	N			
			Would rather not say	R			
		How old were you when you had sexual intercourse for the first time?	*11 years old or younger*	LT11	String	FE	2019
			12 years old	12			
			13 years old	13			
			14 years old	14			
			15 years old	15			
			16 years old	16			
			17 years old	17			
			Over 18 years old	LT18			
			Would rather not say	R			
		The last time you had sexual intercourse did you or your partner use any form of contraception or take any precautions?	*Yes*	Y	String	FE	2019
			No	N			
			Don't know	R			
		Do you feel you can say no to having sexual activity?	*Never*	0-12.5	Sliding scale	FE	2019
			Not often	12.6-37.5			
			Sometimes	37.6-62.4			
			Quite often	62.5-87.4			
			Always	87.5-100			
		Do you know how and where to access contraception and sexual health advice?	*Yes*	Y	String	Sec; FE	2019
			No	N			
		Which of the sexual health and contraception services listed below would you feel comfortable using?			Tick box	FE	2019
		Doctor/GP Surgery		Y			
		Sexual health clinic at hospital		Y			
		School/college drop in		Y			
		School/college nurse		Y			
		Youth centre		Y			
		Pharmacy		Y			

Note: A * in the *Matched* or *Year Included* columns indicates that the *Question* or *Label* column contain differences in the * indicated year or survey version

OxWell 2021 FE

	Supermarket based pharmacy		Y			
	Internet/website		Y			
	C-Card (free condom scheme)		Y			
	None of these					
	Overall, how comfortable would you be using sexual health and contraceptive services?	*Would feel very uncomfortable using these services*	0-12.5	Sliding scale	FE	2019
		Would feel quite uncomfortable using these services	12.6-37.5			
		Would feel OK using these services	37.6-62.4			
		Would feel quite comfortable using these services	62.5-87.4			
		Would feel very comfortable using these services	87.5-100			
	Would you know where to get a condom if you needed one?	*Yes*	Y	String	Sec; FE	2019; 2021*
		No	N			
		Don't Know	R			
	How confident are you about using a condom?	*Not at all confident*	0-12.5	String	FE	2019; 2021
		Not very confident	12.6-37.5			
		Quite confident	37.6-62.4			
		Confident	62.5-87.4			
		Extremely confident	87.5-100			
	If you needed help and advice about contraception/emergency contraception/not getting pregnant or preventing STI's who would you go to for help?			Tick box	Sec; FE	2019; 2021*
	Parent. step-parent or carer		Y			
	Someone else in your family		Y			
	Friend(s)		Y			
	A teacher or other adult at your school/college		Y			
	A school/college nurse		Y			
	A doctor, nurse (outside school/college), psychologist		Y			
	A counsellor, youth worker or social worker		Y			
	Internet advice sites, forums, chatrooms etc		Y			
	No-one		Y			

Note: A * in the *Matched* or *Year Included* columns indicates that the *Question* or *Label* column contain differences in the * indicated year or survey version

OxWell 2021 FE

RESEARCH

CODE	Contingent	Question	Label	Value	Type	Matched	Year Included
		If your answers to this survey could be linked like this to other information on you, would you still take part	*Yes*	Y	String	Prim*; Sec; FE	2021
			No	N			
			Maybe	M			
			I don't understand	D			
		Did you also complete this survey last year at school/college?	*Yes*	Y	String	Prim; Sec; FE	2020; 2021*
			No	N			
			I can't remember	NOMEMORY			
		If your answers to this survey could be linked like this to other information on you, would you like to be able to compare your own answers to the average in your year group?	*Not at all*	0-12.5	Sliding scale	Prim*; Sec; FE	2019*; 2020; 2021*
			Not much	12.6-37.5			
			Some	37.6-62.4			
			Quite a lot	62.5-87.4			
			A great deal	87.5-100			
		If your answers to this survey could be linked to other school/college information on you, would you have answered any of questions in this survey differently?	*Not at all*	0-12.5	Sliding scale	Prim*; Sec; FE	2019*; 2020; 2021*
			Not much	12.6-37.5			
			Some	37.6-62.4			
			Quite a lot	62.5-87.4			
			A great deal	87.5-100			

Note: A * in the *Matched* or *Year Included* columns indicates that the *Question* or *Label* column contain differences in the * indicated year or survey version

OxWell 2021 FE

PARANOIA

CODE	Contingent	Question	Label	Value	Type	Matched	Year Included
		How often have you had these thoughts over the last 2 weeks					
		People at school/college are trying to make me feel unwanted	*Never*	NEVER	String	Sec; FE	2021
			Once	ONCE			
			Couple of times	TWICE			
			Few times a week	FEWTIMES WK			
			Every day	EVERYDAY			
			All the time	CONSTANT			
		I am sure people are gossiping about me on social media	*Never*	NEVER	String	Sec; FE	2021
			Once	ONCE			
			Couple of times	TWICE			
			Few times a week	FEWTIMES WK			
			Every day	EVERYDAY			
			All the time	CONSTANT			
		I am being pushed out of conversations on purpose	*Never*	NEVER	String	Sec; FE	2021
			Once	ONCE			
			Couple of times	TWICE			
			Few times a week	FEWTIMES WK			
			Every day	EVERYDAY			
			All the time	CONSTANT			
		My friends or partner are ignoring my messages to upset me	*Never*	NEVER	String	Sec; FE	2021
			Once	ONCE			
			Couple of times	TWICE			
			Few times a week	FEWTIMES WK			
			Every day	EVERYDAY			
			All the time	CONSTANT			
		People are trying to embarrass me in class on purpose	*Never*	NEVER	String	Sec; FE	2021
			Once	ONCE			
			Couple of times	TWICE			
			Few times a week	FEWTIMES WK			
			Every day	EVERYDAY			
			All the time	CONSTANT			

Note: A * in the *Matched* or *Year Included* columns indicates that the *Question* or *Label* column contain differences in the * indicated year or survey version

OxWell 2021 FE

	People are making sly comments to upset me	*Never*	NEVER	String	Sec; FE	2021
		Once	ONCE			
		Couple of times	TWICE			
		Few times a week	FEWTIMES WK			
		Every day	EVERYDAY			
		All the time	CONSTANT			
	I think people are lying to me on purpose	*Never*	NEVER	String	Sec; FE	2021
		Once	ONCE			
		Couple of times	TWICE			
		Few times a week	FEWTIMES WK			
		Every day	EVERYDAY			
		All the time	CONSTANT			
	People say things under their breath to wind me up	*Never*	NEVER	String	Sec; FE	2021
		Once	ONCE			
		Couple of times	TWICE			
		Few times a week	FEWTIMES WK			
		Every day	EVERYDAY			
		All the time	CONSTANT			
	Nasty tricks are being played on me	*Never*	NEVER	String	Sec; FE	2021
		Once	ONCE	String	Sec; FE	2021
		Couple of times	TWICE			
		Few times a week	FEWTIMES WK			
		Every day	EVERYDAY			
		All the time	CONSTANT			
	People are trying to confuse me on purpose	*Never*	NEVER	String	Sec; FE	2021
		Once	ONCE			
		Couple of times	TWICE			
		Few times a week	FEWTIMES WK			
		Every day	EVERYDAY			
		All the time	CONSTANT			
	Groups of people are planning against me	*Never*	NEVER	String	Sec; FE	2021
		Once	ONCE			
		Couple of times	TWICE			

Note: A * in the *Matched* or *Year Included* columns indicates that the *Question* or *Label* column contain differences in the * indicated year or survey version

OxWell 2021 FE

		Few times a week	FEWTIMES WK			
		Every day	EVERYDAY			
		All the time	CONSTANT			
	People are collecting my information or photos to use against me	*Never*	NEVER	String	Sec; FE	2021
		Once	ONCE			
		Couple of times	TWICE			
		Few times a week	FEWTIMES WK			
		Every day	EVERYDAY			
		All the time	CONSTANT			
	I'm sure people are seeking revenge on me	*Never*	NEVER	String	Sec; FE	2021
		Once	ONCE			
		Couple of times	TWICE			
		Few times a week	FEWTIMES WK			
		Every day	EVERYDAY			
		All the time	CONSTANT			
	I feel like I am being followed or stalked	*Never*	NEVER	String	Sec; FE	2021
		Once	ONCE			
		Couple of times	TWICE			
		Few times a week	FEWTIMES WK			
		Every day	EVERYDAY			
		All the time	CONSTANT			
	I am scared of what strangers will do to me	*Never*	NEVER	String	Sec; FE	2021
		Once	ONCE			
		Couple of times	TWICE			
		Few times a week	FEWTIMES WK			
		Every day	EVERYDAY			
		All the time	CONSTANT			
	People will try to kidnap me	*Never*	NEVER	String	Sec; FE	2021
		Once	ONCE			
		Couple of times	TWICE			
		Few times a week	FEWTIMES WK			
		Every day	EVERYDAY			
		All the time	CONSTANT			

Note: A * in the *Matched* or *Year Included* columns indicates that the *Question* or *Label* column contain differences in the * indicated year or survey version

OxWell 2021 FE

	I could be attacked at any time	*Never*	NEVER	String	Sec; FE	2021
		Once	ONCE			
		Couple of times	TWICE			
		Few times a week	FEWTIMES WK			
		Every day	EVERYDAY			
		All the time	CONSTANT			
	I feel unsafe around people everywhere I go	*Never*	NEVER	String	Sec; FE	2021
		Once	ONCE			
		Couple of times	TWICE			
		Few times a week	FEWTIMES WK			
		Every day	EVERYDAY			
		All the time	CONSTANT			

Note: A * in the *Matched* or *Year Included* columns indicates that the *Question* or *Label* column contain differences in the * indicated year or survey version
